# Plant super-barcode: a case study on genome-based identification for closely related species of *Fritillaria*

**DOI:** 10.1186/s13020-021-00460-z

**Published:** 2021-07-05

**Authors:** Lan Wu, Mingli Wu, Ning Cui, Li Xiang, Ying Li, Xiwen Li, Shilin Chen

**Affiliations:** 1 Key Laboratory of Beijing for Identification and Safety Evaluation of Chinese Medicine, Institute of Chinese Materia Medica, China Academy of Chinese Medical Sciences, Beijing, 100700 China; 2 Institute of Medicinal Plant Development, Chinese Academy of Medical Sciences & Peking Union Medical College, Beijing, 100193 China

**Keywords:** Species identification, Closely related species, Chloroplast genome, Super-barcode, Genome comparison, *Fritillaria*

## Abstract

**Background:**

Although molecular analysis offers a wide range of options for species identification, a universal methodology for classifying and distinguishing closely related species remains elusive. This study validated the effectiveness of utilizing the entire chloroplast (cp) genome as a super-barcode to help identify and classify closely related species.

**Methods:**

We here compared 26 complete cp genomes of ten *Fritillaria* species including 18 new sequences sequenced in this study. Each species had repeats and the cp genomes were used as a whole DNA barcode to test whether they can distinguish *Fritillaria* species*.*

**Results:**

The cp genomes of *Fritillaria* medicinal plants were conserved in genome structure, gene type, and gene content. Comparison analysis of the *Fritillaria* cp genomes revealed that the intergenic spacer regions were highly divergent compared with other regions. By constructing the phylogenetic tree by the maximum likelihood and maximum parsimony methods, we found that the entire cp genome showed a high discrimination power for *Fritillaria* species with individuals of each species in a monophyletic clade. These results indicate that cp genome can be used to effectively differentiate medicinal plants from the genus *Fritillaria* at the species level.

**Conclusions:**

This study implies that cp genome can provide distinguishing differences to help identify closely related *Fritillaria* species, and has the potential to be served as a universal super-barcode for plant identification.

**Supplementary Information:**

The online version contains supplementary material available at 10.1186/s13020-021-00460-z.

## Background

Although many biological studies depend on accurate species identification and delimitation, such as the implement of biodiversity conservation, therapy of disease and the identification of invasive species, taxonomic expertise is collapsing [1, 2]. Morphology-based identification of plant species has remained elusive due to the scarcity and ambiguity of diagnostic characters. Fortunately the advent of molecular markers made an impact on species identification, and undoubtedly has made a substantial contribution to systematics. However, currently none of the available DNA loci work for all species, especially for the closely related species. Moreover, multiple closely related species that occupy the same area have always posed insurmountable barriers to the goal of current highly accurate identification [3]. Therefore, a new method is required in the search for a universal marker for taxon recognition.

The chloroplast (cp) genome is a versatile tool for phylogenetics. During the past decade, there have been many analyses addressing phylogenetic questions at deep nodes based upon the complete sequences of cp genomes [4–6]. As plant biologists enter the era in which comparative genomics promises to address in-depth questions, the inestimable effectiveness of cp genome in systematic studies quickly become clear. The entire cp genome contains approximately as much information as does the COI gene used in animals and it has the potential to provide distinguishing differences that can help molecularly identify closely related species [7]. With advances in high-throughput sequencing, achieving cp genome is easily acquirable at a large-scale with lower costs. This has promoted studies of systematics using cp genome in *Epimedium* [8], *Paris* [9] and *Sanguisorba* [10]. Because of the low discrimination power of general molecular markers in plants and their closely related species, researchers have proposed the entire cp genome as a super-barcode to discriminate closely related species [11].

The bulbs of *Fritillaria* species (called BeiMu, BM) have been used medicinally for more than 2000 years, specifically in the treatment of dry cough and blood-stained sputum. Due to the over exploitation of natural resources, the availability of Chuan BeiMu continues to decline [12]. Currently, most *Fritillaria* species used as Chuan BeiMu are in the list of wild protected species (level 3) in China, and the price of high-quality Chuan BeiMu can be as high as ~ 500$/kg. With the decreased availability and high price, Chuan BeiMu is often adulterated by other cheaper bulbs from other *Fritillaria* species, with a market survey reporting the adulteration rate of Chuan BeiMu to be as high as 20% [13].

Presently, *Fritillaria* bulbs are identified by morphological features [14] and chemical properties [15]. Unfortunately, different species can be morphologically similar and they always have the similar chemical constituents, which make the identification of *Fritillaria* difficult at the species level using traditional methods. Although DNA barcoding provided accurate identification for plants, it is insufficient in the authentication of *Fritillaria* species. Luo and Xiang et al. [16, 17] reported that ITS2 sequence could not provide monophyletic clades for the genus *Fritillaria* at the species level. Meanwhile, Sharifi [18] and TÜRKTAŞ et al. [19] constructed the phylogenetic trees based on the *trnH-psbA* and *trnL-trnF* regions using 22 Iranian *Fritillaria* species and ten Turkey *Fritillaria* species respectively, and the phylogenetic trees showed that it is impossible to distinguish these *Fritillaria* species. Rønsted et al*.* [20] presented the same result based on *matK*, *rpl16*, *trnK*, and ITS sequences for *Fritillaria*. Therefore, these findings demonstrate that the single-locus markers have low resolution for *Fritillaria* due to high sequence similarities.

Compared with the most frequently used and predicted genus-specific DNA barcodes, cp genome contains more variations with a significantly higher resolution of phylogenies, which is valuable to reveal phylogenetic relationships between closely related species [12]. Cp genome has been widely applied in phylogenetic analyses [21–24], plant population studies [25], and plant identification [7]. The phylogenetic tree constructed based on complete cp genomes has a higher supporting rate and discrimination power [26]. Li et al*.* [11] therefore proposed to use the entire cp genome as a super-barcode to accurately identify closely related species.

Here, we compared 26 complete cp genomes, including 18 newly sequenced genome sequences for this study, from ten *Fritillaria* species that are included in the Chinese Pharmacopoeia 2020. We performed a comprehensive analysis of the complete cp genomes of the *Fritillaria* species, which are difficult to be identified by morphology and taxonomy alone. The aims of our study were as follows: (1) to verify the hypothesis whether super-barcode can be used as a universal barcode to identify closely related species, (2) to present 18 new complete cp genomes from ten *Fritillaria* species and explore polymorphic regions within *Fritillaria* cp genomes, and (3) to evaluate the discrimination power of cp genomes in the genus *Fritillaria* at the species level. The results demonstrated that the cp genome could be used to identify *Fritillaria* at species level. The entire cp genome was found to be a most promising universal DNA marker in identification of closely related species.

## Methods

### DNA extraction

Twenty-six cp genomes from ten *Fritillaria* species were used in this study (see Additional file 1: Table S1). Fresh leaves of 18 individuals from nine *Fritillaria* species were collected. The cp genomes of eight additional individuals were downloaded from GenBank. Total genomic DNA of each sample was isolated from ~ 200 mg of fresh leaf using the DNeasy Plant Mini Kit (QIAGEN, Germany), according to manufacturer’s instructions. To meet the quality requirements for sequencing, we assessed the quality and quantity of each DNA sample using a Qubit2.0 Fluorometer (Thermo Scientific, USA) and a NanoDrop 2000 Spectrophotometer (Nanodrop Technologies, Wilmington, DE, USA), respectively.

### Genome sequencing, assembly and annotation

The shotgun libraries (450 bp) were constructed using ~ 2 μg of total DNA according to the manufacturer’s instructions (Illumina Inc., San Diego, CA). A total of 11 cp genomes from seven *Fritillaria* species were sequenced using Illumina HiSeq X platform (Illumina, San Diego, CA, USA), and we obtained > 2 Gb data for each sample. Raw reads were filtered using the Fastqc trim tool (http://www.bioinformatics.bbsrc.ac.uk/projects/fastqc). Thereafter, contigs were extracted by BLASTs [27], and the cp genomes of six published *Fritillaria* species (Accession No.: KF769143, KF712486, KY646166, KC713823, KF769142, and KY646165) were set as reference sequences. The contigs were assembled using SOAPdenovo [28]. Sequence extension was performed using SSPACE [29], and the gap fillers were excluded by GapCloser [30]. Other seven cp genomes from six *Fritillaria* species were sequenced using Roche 454 titanium sequencing platform and assembled using Newbler sequence assembler. The four junctions between IRs (inverted repeats) and SC (large single-copy region, LSC; small single-copy region, SSC) were validated by PCR amplification and Sanger sequencing with specific primers, as listed in Additional file 2: Table S2. The initial gene annotation was performed using CpGAVAS [31]. Identified tRNA genes were confirmed by tRNAscan-SE [32, 33]. Circular cp genome maps were drawn using OGDRAW software (http://ogdraw.mpimp-golm.mpg.de/) [34]. GC content was analyzed using MEGA5.0. [35]. The validated entire 18 cp genome sequences were deposited in NCBI (Accession No. were listed in Additional file 1: Table S1).

### Genome comparison and divergent analyses

Comparison of the sequence divergence in the cp genomes of ten *Fritillaria* species was performed using the mVISTA [36, 37] program in the Shuffle-LAGAN mode, and the annotation of *F. unibracteata* (MN148410) was used as the reference. In addition, simple sequence repeats (SSRs) were detected using MISA (http://pgrc.ipk-gatersleben.de/misa/) [38] with thresholds of repeat numbers of eight, four, four, three, three and three for mono-, di-, tri-, tetra-, penta- and hexa-nucleotides, respectively. Then, insertions/deletions (indels) were counted using LASTZ software and single nucleotide polymorphisms (SNPs) were analysed by MUMmer. All SNPs in the coding sequence were detected whether it affects the protein sequence and were distinguished from synonymous and non-synonymous SNPs. Variations were visualized by Circos software [39] including A-G layers. The discrimination ability of highly variable loci selected in this study was tested using 26 samples from 10 species.

### Species identification

To evaluate the effectiveness of super-barcode in identification for closely related species, 26 complete cp genome sequences were aligned using the MAFFT program [40], and then adjusted manually in Bioedit. Phylogenetic trees were constructed by the maximum likelihood (ML) and maximum parsimony (MP) methods. *Lilium brownie* (accession no.: KY748296) and *Cardiocrinum giganteum* (Accession No.: KX528334) were set as outgroups. ML analyses were conducted using RAxML-HPC2 on XSEDE at the CIPRES Science Gateway website (https://www.phylo.org/) [41] with the GTR + I + G model as the best-fitting model, which was selected by jModelTest 2.1.4 [42]. MP analyses were performed using PAUP*4.0b10 [43]. The branch support of the tree was estimated in 1000 bootstrap replicates.

## Results

### Genome features

**Fig. 1:**
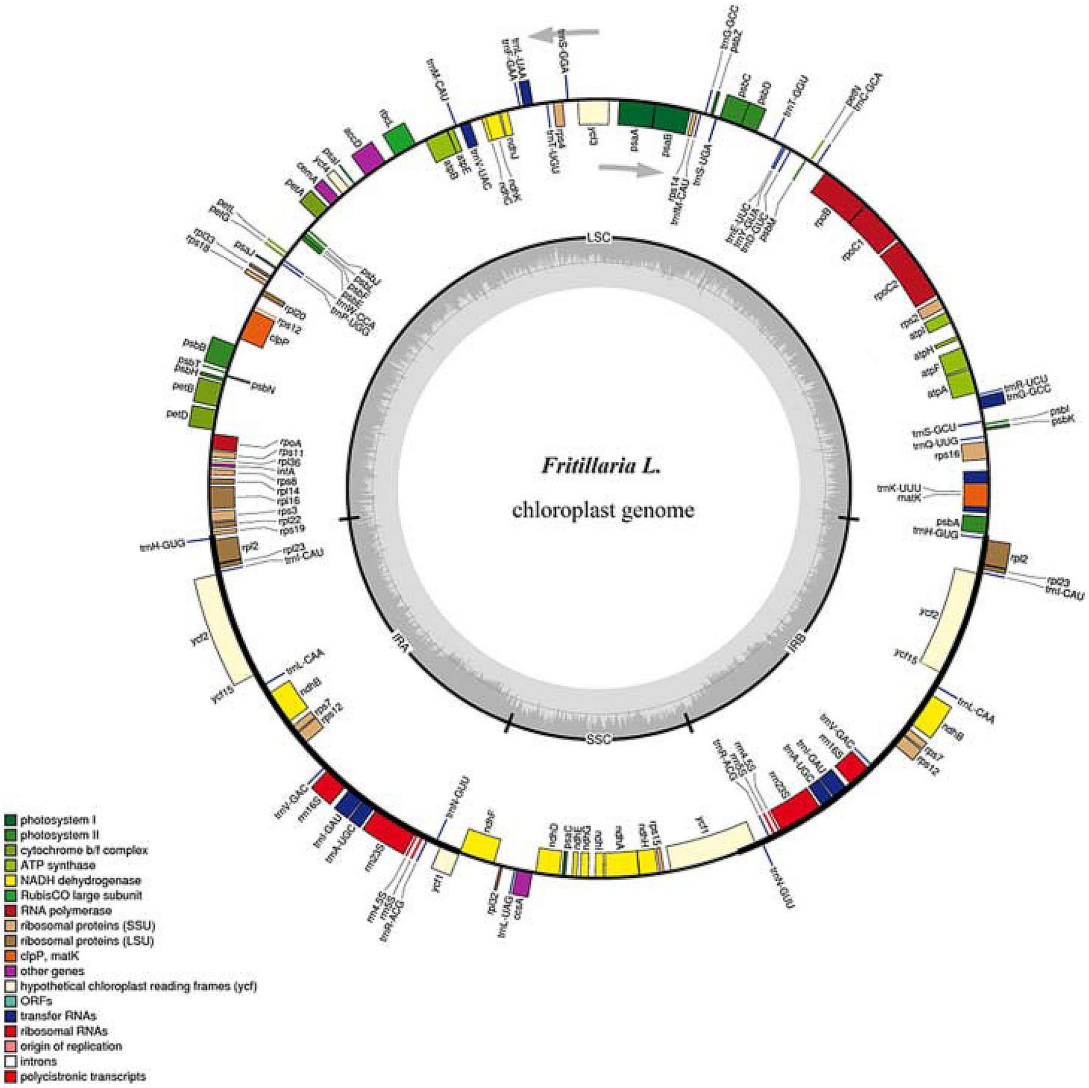
Gene map based on 26 *Fritillaria* cp genomes. Genes shown outside and inside the circle are transcribed counterclockwise and clockwise, respectively. Different functional gene groups are color-coded

All the 26 cp genomes were similar in length, among which the shortest was *F. unibracteata* (150,764 bp) and the longest was *F. hupehensis* (152,186 bp), with the typical quadripartite structure of angiosperms. They contained a LSC (81,182–81,926 bp) and a SSC (17,114–17,586 bp), separated by a pair of IRs (26,024–26,390 bp) (see Fig. 1 and Additional file 1: Table S1). The GC content was unevenly distributed throughout *Fritillaria* cp genomes. In the *F. cirrhosa* (MN148400) cp genome, for example, GC content of the IR region (42.5%) was significantly higher than that of the LSC region (34.7%) or the SSC region (30.5%). This may be a reason that the conservation is divergent between the IR and LSC/SSC regions [10, 44]. The coding regions accounted for 52.5% of the genome, and therefore, the non-coding regions, such as the pseudogenes, introns, and intergenic spacers, accounted for 47.5%. The 26 *Fritillaria* cp genomes possessed 114 unique genes (Fig. 1) that included 80 protein-coding genes, 30 tRNA genes and four rRNA genes. In addition, we identified two pseudogenes (*infA* and *ycf15*). The *rps12* is a trans-spliced gene in which two 3′ end residues are located within the IR region and the 5′ end is located within the LSC region.

Introns are critical for the regulation of alternative splicing in the genome [45]. Similar to other angiosperm [16, 46], we identified 18 intron-containing genes in each of the 26 *Fritillaria* cp genomes, which included 12 protein-coding genes and six tRNA genes. Fifteen out of the 18 genes contained a single intron, whereas the remaining three genes (*ycf3*, *clpP*, and *rps12*) contained two introns.

### SSR analysis

SSRs are short (1–6 nucleotide repeat units) tandemly repeated sequences that are widely distributed across the entire cp genome, and they are important for the population studies in plants. We here analyzed the distribution of SSRs in 26 *Fritillaria* cp genomes. The number of SSRs ranged from 179 in *F. cirrhosa* to 195 in *F. pallidiflora*. Most of the SSRs were mononucleotide repeats ranging from 113 in *F. unibracteata* to 122 in *F. hupehensis*. The number of di-, tri- and tetranucleotide repeats was 57–63, 1–5 and 6–10, respectively. The number of penta- and hexanucleotide repeats were few, and none were detected in most *Fritillaria* cp genomes.

### Comparative analysis of cp genome

Using the *F. unibracteata* (MN148410) as reference, cp genomes of ten *Fritillaria* species were compared and analyzed to show the sequence divergence, which is relevant to further phylogeny and species authentication analyses. The genome comparison showed that there was a high similarity among these cp genomes. The sequences in LSC regions were more divergent than in the SSC and IR regions. The high divergences occurred in *trnS-GCU-trnG-GCC, trnG-GCC-trnR-UCU, trnE-UUC-trnT-GGU, trnT-GGU-psbD, atpH-atpI, trnT-UGU-trnL-UAA* and *psbE-petL* (see Additional file 3: Figure S1). The identification efficien of these seven loci were tested in this study and data showed that they could not distinguish all 26 samples from ten *Fritillaria* species (see Additional file 4: Table S3).

**Fig. 2:**
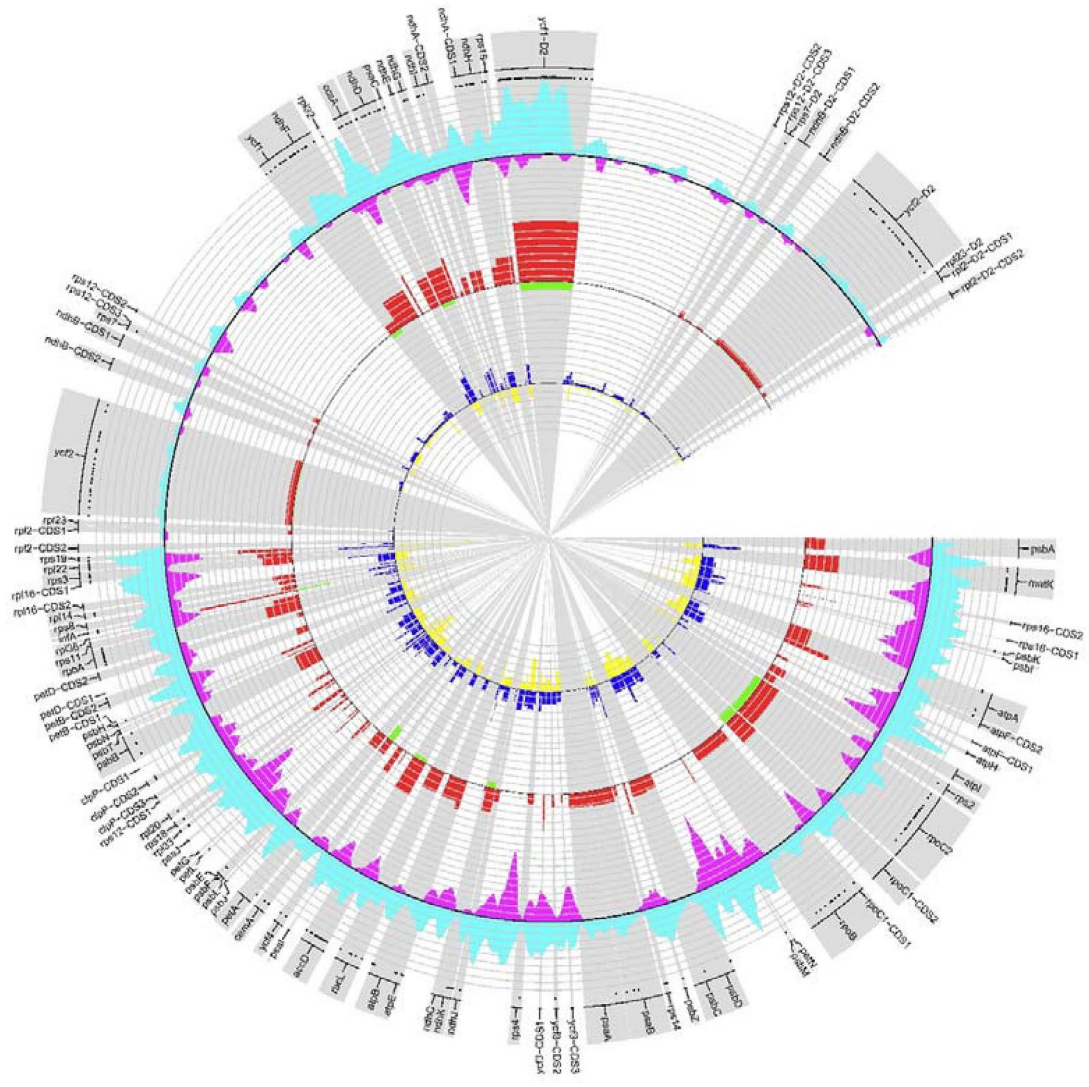
SNP and indel variation among cp genomes of *Fritillaria*. Track A represent nonsynonymous SNP occurrence in genes. Track B shows identified SNPs (cyan histogram) while track C represents identified indels (magenta histogram), with 100 bp shift per 600 bp window size. Track D and E represent percent of SNPs and indels per CDS length, respectively. Track F and G show percent of SNPs and indels per noncoding region length, respectively

In addition, sequence variability was estimated using SNPs and indels (Fig. 2). Based on cp genome-wide investigations, a total of 2449 SNPs and 565 Indels were detected among ten *Fritillaria* species (Table 1). Most of the variants (838 SNPs and 358 Indels) were located in intergenic spacers. Analysis of the distribution of genetic variability revealed that the most variable protein-coding region was the *rps19* gene. It is located in the Large Single Copy with a length of 279 bp containing 10 SNP sites. Among the non-coding regions, the highest frequency of polymorphism was found in the *rpl22-rps19* spacer. Within the 127 bp-long *rpl22-rps19* region, 14 SNPs and 10 indels were identified. Relatively high variability was also characteristic for the *rpl16-rps3* spacer.

**Table 1:** The SNP and Indels in 26 cp genomes of the ten *Fritillaria* species

Region	Coding region		Intergenic spacer		Intron		Summary	
	SNP	InDel	SNP	InDel	SNP	InDel	SNP	InDel
LSC	600	14	838	358	254	87	1692	459
SSC	383	10	107	29	31	16	521	55
IR_a_	47	3	60	20	12	6	119	29
IR_b_	45	2	60	15	12	5	117	22
Total	1075	29	1065	422	309	114	2449	565

Nowadays, cp DNA regions have been widely used in studies on species identification and phylogenetic analysis. However, none of cp markers work for *Fritillaria* species in previous studies [47, 48] as well as in this study.

### Species identification

In this study, MP and ML trees were constructed based on 26 complete cp genomes from ten *Fritillaria* species with two species (*Lilium brownie* and *Cardiocrinum giganteum*) from the same family as outgroup. Our results showed that phylogenetic trees constructed by the entire cp genome presented a high discrimination power for the *Fritillaria* species, and different individuals in each species were resolved as a monophyletic clade. At first, *Fritillaria* medicinal plants were divided into two large branches, and *F. ussuriensis* was separated out with an approval rate of 100%. Thereafter, *F. walujewii* and *F. pallidiflora* were integrated into one big branch and separated from the other *Fritillaria* medicinal plants, with a supporting rate of 100%. These two medicinal species were used as Fritillariae pallidiflorae bulbus (Yi BeiMu), which was recorded in the Chinese Pharmacopoeia 2020. In addition, individuals of these two species were separated into two monophyletic clades. Lastly, the original species of Fritillariae cirrhosae bulbus (Chuan BeiMu) were gathered into one branch and separated from the other two types of *Fritillaria* bulbs, which included Fritillariae thunbergii bulbus (Zhe BeiMu) and Fritillariae hupehensis bulbus (Hubei BeiMu), with an approval rate of 100%. In addition, individuals of all species from Fritillariae cirrhosae bulbus (Chuan BeiMu) were separated into a monophyletic clade for each species, respectively. For the five types of *Fritillaria* bulbs, Fritillariae cirrhosae bulbus (Chuan BeiMu) had the closest relationship with Fritillariae thunbergii bulbus (Zhe BeiMu) and Fritillariae hupehensis bulbus (Hubei BeiMu). In total, all individuals of the original species from each *Fritillaria* bulbs recorded in the Chinese Pharmacopoeia 2020 were clustered and separated from other *Fritillaria* bulbs with a high branch supporting rate.

Ten *Fritillaria* species covered five different types of traditional medicinal materials: “PingBeiMu (PBM)” which original plant species is from *F. ussuriensis*, “ChuanBeiMu (CBM)” which original plant species are from *F. unibracteata var. wabuensis, F. unibracteata, F. taipaiensis, F. cirrhosa* and *F. delavayi, “*ZheBeiMu (ZBM)*”* from *F. thunbergii*, “HuBeiBeiMu (HBBM)” from *F. hupehensis*, “YiBeiMu (YBM)” from *F. pallidiflora* and *F. walujewii*. Phylogenetic trees in this study formed into five groups which branches were drawn in different colors. We found that the five monophyletic clades were consistent with five types of BM material medica. Another interesting finding is that the topologies of phylogenetic trees also formed into five major groups: PBM, ZBM, HBBM, CBM, and YBM. The five groups belong to five different ecological and geographical regions **(**see Additional file 6: Figure S3**)**. PBM is mainly distributed in the plain of Mid-temperate zone in Northeast China. ZBM is mainly distributed in the subtropical plain and close to the ocean. HBBM is mainly loacted in plain mountain with a subtropical climate. CBM is mainly located in plateau mountain of western China, and YBM is mainly distributed in Mid-temperate zone of western plateau.

## Discussion

This study investigated the feasibility of developing a cp-genome based identification method for closely related plants at lower taxonomic levels. Although DNA barcoding provides accurate identification for plants, it remains a significant challenge for authentication of *Fritillaria* species. Firstly we analyzed the cp genome of *Fritillaria.* They were highly conserved in gene structure, gene order and gene content. The average GC content was ~ 37.0%, which was similar to the published cp genomes of Liliales species [48, 49]. We also investigated introns in 26 *Fritillaria* cp genomes. In this study, most of the protein-coding genes had the standard ATG as the initiator codon, but *rpl2*, *ndhD* and *rps19* genes started with AUG, ATC and GUG, respectively. This variation, which may have been caused by RNA editing [50], has been reported in other cp genomes as well [51, 52]. As valuable molecular markers, SSRs are widely used in studies of population genetics, molecular breeding and species identification because of high polymorphisms [53, 54]. In this study, the distribution of SSRs in *Fritillaria* cp genomes was different but the interspecific variations were higher than intraspecific variations. In addition, most SSRs were located in the LSC. The content of polyA/T was greater than that of polyG/C. We speculate that the richness of A/T SSRs may be related to the AT abundance in these *Fritillaria* genomes [22, 55]. Comparative analysis showed a high similarity among 26 *Fritillaria* cp genomes.

In order to find ideal molecular markers in *Fritillaria*, many studies tried to select highly variable regions based on cp genomes as genus-specific DNA barcodes for species identification [56–64]. Li et al. [56] found eight genes which had abundant variations among species by comparing four *Fritillaria* cp genomes. And Li et al. [57] performed multiple sequence alignment analysis on gene and intergenic regions respectively using clustalw2 and chose 20 highly variable genes and 20 highly variable intergenic regions. They found that both genes and intergenic regions in *Fritillaria* were relatively conservative compared with other species. Only seven hypervariable intergenic regions were selected as potential specific DNA barcode based on comparison of four *Fritillaria* cp genomes. Similar results existed in other studies [58–64], except that the number of highly variable regions was different. Unfortunately, none of the above regions have been verified by further experiments. We tested all 57 highly variable loci selected by published works except regions over 2 k bp in length using 26 samples from 10 *Fritillaria* species (see Additional file 4: Table S3 and Additional file 7). A total of seven hypervariable sites were screened and verified in this study. We found none of the loci could identify all these 10 species (see Additional file 4: Table S3). Therefore, these findings demonstrated that traditional molecular methods including DNA barcoding could not solve the problem of species identification in *Fritillaria* due to high sequence similarities.

Because of the low discriminatory ability of general molecular markers in plants and their closely related species, researchers have placed high hopes on the use of plastid genome sequences in plant identification [7, 65, 66]. Some authors have performed tentative studies to test the potential of cp genomes in certain plant groups of closely related species. Bayly et al. [67] presented a phylogenetic analysis in three genera (*Eucalyptus*, *Corymbia* and *Angophora*) and demonstrated that cp genome was useful in lower-level genetic studies. Yang et al. [68] found that the cp genome lighted the species identification as organelle scale-scale “barcodes”. Li et al. [11] then put forward that cp genome can be regarded as a super-barcode for closely related species. Xia et al. [69] and Chen et al. [70] tested the ability of super-barcode in *Chrysanthemum* and *Ligularia* respectively. However, the above two studies lacked sufficient species number and intraspecific samples. We here extended earlier investigations on a large scale to evaluate the feasibility of using the cp-genome to discriminate closely related species of *Fritillaria*.

**Fig. 3:**
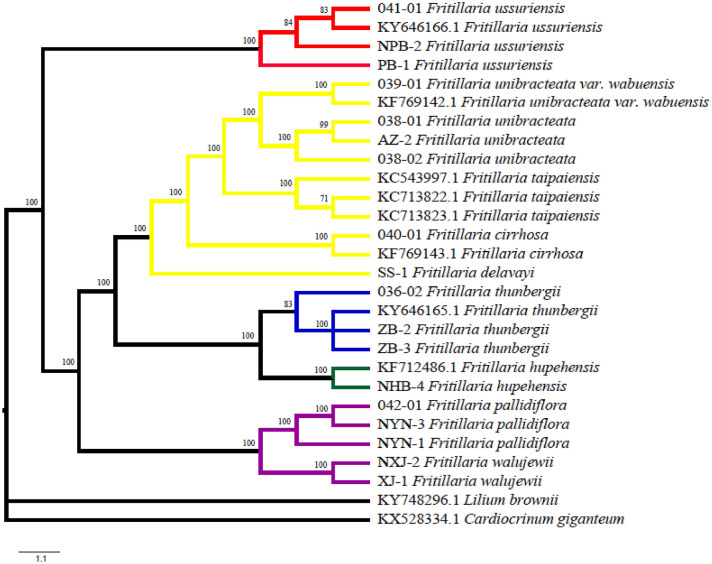
Phylogenetic relationships among the ten *Fritillaria* species based on complete cp genome sequences by the maximum parsimony (MP) method. *Lilium brownie* and *Cardiocrinum giganteum* were set as the compound outgroups

Our results showed that the two topologies of MP and ML were identical with high support values (see Fig. 3 and Additional file 5: Figure S2). Both phylogenetic trees constructed by the entire cp genome presented a high discrimination power for the *Fritillaria* species, and different individuals from same species were formed into a monophyletic clade whatever in species level and in subspecies level. The cp-genome possesses the basic qualifications to be a universal marker compared with traditional molecular identification markers. First, Chloroplasts are haploid and non-recombining and cp-genome sequences are highly conserved, so they can act as a single locus [66]. Second, in contrast to a single gene, they have more variation and have the potential to identify closely related species at lower taxonomic levels [7]. Third, in our study, chloroplast sequence data has really shown 100% identification efficiency in *Fritillaria*. Because the results of screening genus-specific barcodes with different species groups were distinct, plant identification of closely related species based on super-barcode using DNA barcoding may no longer need to choose between more loci or more taxa.

Some authors disagreed with this approach for several reasons, including high expenditures, compared to Sanger sequencing, and the lack of close reference sequences for assembly [71]. With the development of next generation sequencing, the cost for cp-genome sequencing and assembly is almost the same as that of PCR-based sequencing on average. And for most plants, close reference for assembly has become less important than before. Taking the entire cp genome as a super-barcode becomes feasible for accurate species identification, since it has been demonstrated that cp genome could provide a higher resolution in species authentication in species and even population level [7, 25, 26].

Our results showed that the super-barcode based on the full length of the cp genome sequence could successfully distinguish the *Fritillaria* species recorded in the Chinese Pharmacopoeia 2020. According to the pharmacophylogenetic theory of Xiao et al. [72], the species that had the closest phylogenetic relationships were also similar in chemical components and curative effects. This confirmed the accuracy and reliability of the original species division for the five types of *Fritillaria* bulbs in the Chinese Pharmacopoeia 2020. Furthermore, this study demonstrated that the super-barcode based on the full length of the *Fritillaria* cp genome sequence could not only successfully distinguish the *Fritillaria* species in species level but also reflect the characteristics of biogeography. The analysis of phylogenetic relationship was coincident with geographical distribution of BM (see Additional file 6: Figure S3), which provided a way for alternative resource discovery in natural drug development.

Although super-barcode has many advantages, it is not suitable for plant species identification when DNA extraction is difficult. For dried, cooked or decocting material medica, DNA is degraded seriously. We may not extract enough DNA or longer DNA fragments. It is not easy to obtain whole cp genome sequence by assembly. Compared with a single-locus barcode, the cost of super-barcode is higher and data analysis is complex using windows software. In fact, we don’t recommend superbarcode if commonly used DNA barcode can make accurate identification. Therefore super-barcode is a useful supplement to the current molecular identification. It can show its advantages when traditional DNA barcoding is limited to plant identification of some closely related species.

## Conclusions

The cp genome is now a reasonable option for increasing the resolution of plant identification in closely related species. In this study, we firstly verified the hypothesis that the cp genome could be used as a super-barcode to actually identify closely related species. Secondly, we analyzed and compared 26 complete cp genomes of the ten *Fritillaria* species, including 18 newly sequenced genomes. Finally, the phylogenetic analysis constructed by the 26 complete cp genomes strongly showed that the medicinal plants from the genus *Fritillaria* can effectively be distinguished at the species level. Recent advances in sequencing strategies make an unprecedented depth and scale of plastid genome sampling possible. Plastome sequencing is now a reasonable option for increasing the resolution of plant identification studies at low taxonomic levels. We are encouraged by the fact that species identification based upon the cp-genome is generally straightforward. Although there are some issues to be solved, i.e., intraspecies sampling remains sporadic, and discrimination is not rapid, we believe that super-barcode is a good choice for identification of closely related species especially when DNA barcoding encounters difficulties.

## Supplementary Information

**Additional file 1: Table S1.** Sequence information of 26 individuals from ten *Fritillaria* species cp genomes.

**Additional file 2: Table S2**. Specific primers used for validation in assembly.

**Additional file 3: Figure S1.** Comparison of the ten *Fritillaria* species cp genomes using mVISTA.

**Additional file 4: Table S3.** Species resolution of selected highly variable regions in related literatures and this study.

**Additional file 5: Figure S2**. Phylogenetic relationships among the ten *Fritillaria* species based on complete cp genome sequences by the maximum likelihood (ML) method. *Lilium brownie* and *Cardiocrinum giganteum* were set as the compound outgroups.

**Additional file 6: Figure S3**. Ecological and geographical regions of five BM material medica.

**Additional file 7: **Verification of discrimination ability of 57 highly variable loci selected by published works.

## Data Availability

All data generated in this study has been submitted to the NCBI under the following Accession Numbers: MN148400-MN148416, and MN126570.

## Abbreviations

IRs: Inverted repeats regions
ITS/ITS2: Internal transcribed spacer/2
SSC: Small single-copy regions
SSRs: Simple sequence repeats
SNPs: Single nucleotide polymorphisms
LSC: Large single-copy regions
ML: Maximum likelihood
MP: Maximum parsimony
NJ: Neighbour-joining

## Declarations

Prof. Shilin Chen serves as Associate Editor of *Chinese Medicine*.
