# Supplementary material for: Plant super-barcode: a case study on genome-based identification for closely related species of Fritillaria
Source: Chin Med. 2021 Jul 5;16:52. doi: 10.1186/s13020-021-00460-z (PMC8256587; doi:10.1186/s13020-021-00460-z)
Supplement: Supplementary file 1 — Additional file 1: Table S1. Sequence information of 26 individuals from ten Fritillaria species cp genomes. [file 13020_2021_460_MOESM1_ESM.docx]

Plant Super-barcode: A Case Study on Genome-based Identification for Closely Related Species of *Fritillaria*

Lan Wu^1†^, Mingli Wu^1†^, Ning Cui^1^, Li Xiang^1^, Ying Li^2^, Xiwen Li^1*^, Shilin Chen^1*^

^1^Key Laboratory of Beijing for Identification and Safety Evaluation of Chinese Medicine, Institute of Chinese Materia Medica, China Academy of Chinese Medical Sciences, Beijing 100700, China.

^2^Institute of Medicinal Plant Development, Chinese Academy of Medical Sciences & Peking Union Medical College, Beijing 100193, China

^*^Correspondence: [xwli@icmm.ac.cn](mailto:xwli@icmm.ac.cn); slchen@icmm.ac.cn.

^†^Lan Wu and Mingli Wu contributed equally to this work.

Additional file 1: Table S1. Sequence information of 26 individuals from ten *Fritillaria* species cp genomes.

Additional file 2: Table S2. Specific primers used for validation in assembly.

Additional file 3: Figure S1. Comparison of the ten *Fritillaria* species cp genomes using mVISTA.

Additional file 4: Table S3. Species resolution of selected highly variable regions in related literatures and this study.

Additional file 5: Figure S2. Phylogenetic relationships among the ten *Fritillaria* species based on complete cp genome sequences by the maximum likelihood (ML) method. *Lilium brownie* and *Cardiocrinum giganteum* were set as the compound outgroups.

Additional file 6: Figure S3. Ecological and geographical regions of five BM material medica.

Additional file 7：Verification of discrimination ability of 57 highly variable loci selected by published works.

**Additional file 1: Table S1**. **Sequence information of 26 individuals from ten *Fritillaria* species cp genomes.**

|  | TCM |  | Cp genome length | GC content | LSC | SSC | IRs |  |  |
| --- | --- | --- | --- | --- | --- | --- | --- | --- | --- |
| No. | (pinyin) | Species | (bp) | (%) | (bp) | (bp) | (bp) | Voucher No. | Accession No. |
| 1 | Chuan BeiMu | *F. cirrhosa* | 152,001 | 37.0 | 81,773 | 17,532 | 26,348 | 040-01^b^ | MN148400 |
| 2 |  | *F. cirrhosa* | 151,991 | 36.9 | 81,769 | 17,535 | 26,344 |  | KF769143^c^ |
| 3 |  | *F. unibracteata* | 151,067 | 37.0 | 81,388 | 17,537 | 26,071 | AZ-2^a^ | MN148410 |
| 4 |  | *F. unibracteata* | 150,764 | 37.0 | 81,182 | 17,534 | 26,024 | 038-01^b^ | MN126570 |
| 5 |  | *F. unibracteata* | 150,993 | 37.0 | 81,279 | 17,530 | 26,092 | 038-02^b^ | MN148409 |
| 6 |  | *F. delavayi* | 151,753 | 37.0 | 81,469 | 17,586 | 26,349 | SS-1^a^ | MN148401 |
| 7 |  | *F. taipaiensis* | 151,693 | 37.0 | 81,437 | 17,552 | 26,352 |  | KC543997^c^ |
| 8 |  | *F. taipaiensis* | 151,682 | 37.0 | 81,428 | 17,548 | 26,353 |  | KC713822^c^ |
| 9 |  | *F. taipaiensis* | 151,691 | 37.0 | 81,437 | 17,550 | 26,352 |  | KC713823^c^ |
| 10 |  | *F. unibracteata* var. *wabuensis* | 150,942 | 37.0 | 81,223 | 17,535 | 26,092 | 039-01^b^ | MN148411 |
| 11 |  | *F. unibracteata* var. *wabuensis* | 151,009 | 37.0 | 81,286 | 17,541 | 26,091 |  | KF769142^c^ |
| 12 | Yi BeiMu | *F. walujewii* | 151,950 | 37.0 | 81,733 | 17,521 | 26,348 | XJ-1^a^ | MN148416 |
| 13 |  | *F. walujewii* | 151,928 | 36.9 | 81,710 | 17,520 | 26,349 | NXJ-2^a^ | MN148415 |
| 14 |  | *F. pallidiflora* | 152,094 | 37.0 | 81,807 | 17,537 | 26,375 | NYN-1^a^ | MN148404 |
| 15 |  | *F. pallidiflora* | 152,072 | 37.0 | 81,778 | 17,514 | 26,390 | NYN-3^a^ | MN148405 |
| 16 |  | *F. pallidiflora* | 152,028 | 37.0 | 81,728 | 17,522 | 26,389 | 042-01^b^ | MN148403 |
| 17 | Zhe BeiMu | *F. thunbergii* | 152,159 | 37.0 | 81,894 | 17,565 | 26,350 | ZB-2^a^ | MN148407 |
| 18 |  | *F. thunbergii* | 152,158 | 37.0 | 81,894 | 17,564 | 26,350 | ZB-3^a^ | MN148408 |
| 19 |  | *F. thunbergii* | 152,121 | 37.0 | 81,856 | 17,547 | 26,359 | 036-02^b^ | MN148406 |
| 20 |  | *F. thunbergii* | 152,155 | 37.0 | 81,890 | 17,565 | 26,350 |  | KY646165^c^ |
| 21 | Ping BeiMu | *F. ussuriensis* | 151,501 | 37.0 | 81,718 | 17,123 | 26,330 | PB-1^a^ | MN148414 |
| 22 |  | *F. ussuriensis* | 151,540 | 37.0 | 81,746 | 17,126 | 26,334 | NPB-2^a^ | MN148413 |
| 23 |  | *F. ussuriensis* | 151,440 | 37.0 | 81,658 | 17,122 | 26,330 | 041-01^b^ | MN148412 |
| 24 |  | *F. ussuriensis* | 151,524 | 37.0 | 81,734 | 17,114 | 26,338 |  | KY646166^c^ |
| 25 | Hubei BeiMu | *F. hupehensis* | 152,186 | 37.0 | 81,926 | 17,556 | 26,352 | NHB-4^a^ | MN148402 |
| 26 |  | *F. hupehensis* | 152,145 | 37.0 | 81,894 | 17,553 | 26,349 |  | KF712486^c^ |

^a^ sequenced on the Illumina HiSeq X platform in this study

^b^ sequenced with the Roche 454 [titanium sequencing platform](https://journals.plos.org/plosone/article?id=10.1371/journal.pone.0025263) in this study

^c^ cp genomes downloaded from GenBank
