# Supplementary material for: Plant super-barcode: a case study on genome-based identification for closely related species of Fritillaria
Source: Chin Med. 2021 Jul 5;16:52. doi: 10.1186/s13020-021-00460-z (PMC8256587; doi:10.1186/s13020-021-00460-z)
Supplement: Supplementary file 2 — Additional file 2: Table S2. Specific primers used for validation in assembly. [file 13020_2021_460_MOESM2_ESM.docx]

**Additional file 2: Table S2. Specific primers used for validation in assembly.**

| Marker | Primers | Sequence（5’-3’） | Bases | Tm（℃） | GC  % | Annealing temperature（℃） |
| --- | --- | --- | --- | --- | --- | --- |
| *trnS*-*rps4* | *trnS*（F） | AGGCGTAGCATTGGAACT | 18 | 52.7 | 50 | 50 |
|  | *rps4*（R） | AAAATAAATGAGTTGTTAGTCGTAG | 25 | 53 | 28 |  |
| *trnD*-*trnY* | *trnD*（F） | CTACAATCCCAGCGAGGT | 18 | 52.7 | 55.6 | 50 |
|  | *trnY*（R） | CTACGCTGGTTCAAATCC | 18 | 50.7 | 55 |  |
| *rpoB*-*trnC* | *rpoB*（F） | CTGGTATTGTGGACATTTCCTCATT | 25 | 61.5 | 40 | 55 |
|  | *trnC*（R） | GTCAATCAGGCGACACCC | 18 | 56 | 61.1 |  |
| *psaJ*-*rpl33* | *psaJ*（F） | CCTGTGCTAAGTACTCTATGGTTTG | 25 | 57.8 | 44 | 53 |
|  | *rpl33*（R） | TTCGGGCACAACAACAAG | 18 | 55.4 | 50 |  |
| *trnE*-*trnT* | *trnE*（F） | CTCCTTGAAAGAGAGATGTCCTGAA | 25 | 61.2 | 44 | 53 |
|  | *trnT*（R） | ATGGCGTTACTCTACCACTGA | 21 | 54.9 | 47.6 |  |
| *ndhF*-*rpl32* | *ndhF*（F） | GTTGCTATAGGAATAAACAGAAGTC | 25 | 54.5 | 36 | 49 |
|  | *rpl32*（R） | GAATGCCCAGTAGAAACC | 18 | 49.5 | 50 |  |
| *trnP*-*psaJ* | *trnP*（F） | GAAGTAATAGGTAGGGATGACAGGA | 25 | 58.3 | 44 | 56 |
|  | *psaJ*（R） | CGCTAAAGACCCAAACCATAGAGTA | 25 | 61.6 | 44 |  |
