# Supplementary material for: Plant super-barcode: a case study on genome-based identification for closely related species of Fritillaria
Source: Chin Med. 2021 Jul 5;16:52. doi: 10.1186/s13020-021-00460-z (PMC8256587; doi:10.1186/s13020-021-00460-z)
Supplement: Supplementary file 3 — Additional file 3: Figure S1. Comparison of the ten Fritillaria species cp genomes using mVISTA. [file 13020_2021_460_MOESM3_ESM.docx]

**
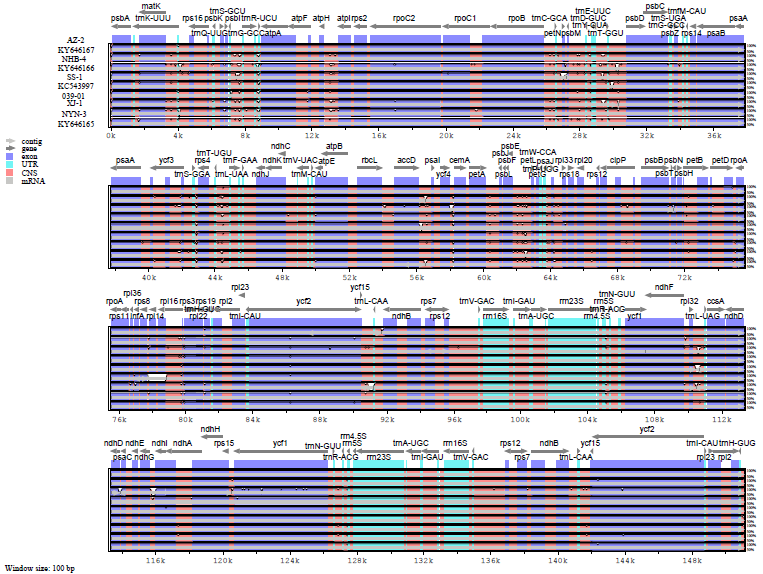
Additional file 3: Figure S1.** Comparison of the ten *Fritillaria* species cp genomes using mVISTA. Grey arrows above the alignments represent the gene orientation. The CNS represents conserved noncoding sequences. The X-scale indicates the location in the cp genome. The Y-scale indicates the percent identity from 50% to 100%.
