# Supplementary material for: Plant super-barcode: a case study on genome-based identification for closely related species of Fritillaria
Source: Chin Med. 2021 Jul 5;16:52. doi: 10.1186/s13020-021-00460-z (PMC8256587; doi:10.1186/s13020-021-00460-z)
Supplement: Supplementary file 4 — Additional file 4: Table S3. Species resolution of selected highly variable regions in related literatures and this study. [file 13020_2021_460_MOESM4_ESM.docx]

**Additional file 4: Table S3.** **Species resolution of selected highly variable regions in related literatures and this study.**

|  | Studies | Hypervariable DNA regions | Species resolution* (%) | Indistinguishable *Fritillaria* species |
| --- | --- | --- | --- | --- |
| 1 | This study | *trnG-trnR* | 30 | *F. unibracteata* var. *wabuensis, F. unibracteata, F. hupehensis, F. pallidiflora, F. cirrhosa, F. taipaiensis, F. thunbergii* |
|  |  | *rpl22-rps19* | 50 | *F. unibracteata* var. *wabuensis, F. unibracteata, F. hupehensis, F. pallidiflora, F. thunbergii* |
|  |  | *trnS-trnG* | 60 | *F. unibracteata* var*. wabuensis, F. unibracteata, F. hupehensis, F. thunbergii* |
|  |  | *trnE-trnT* | 70 | *F. unibracteata* var*. wabuensis, F. thunbergii, F. pallidiflora* |
|  |  | *trnT-psbD* | 80 | *F. unibracteata* var. *wabuensis, F. unibracteata* |
|  |  | *atpH-atpI* | 90 | *F. unibracteata* var. *wabuensis* |
|  |  | *trnT-trnL* | 80 | *F. unibracteata* var*. wabuensis, F. unibracteata* |
|  |  | *psbE-petL* | 80 | *F. unibracteata* var*. wabuensis, F. unibracteata* |
|  |  | *rps19* | 40 | *F. unibracteata* var. *wabuensis, F. unibracteata, F. pallidiflora, F. walujewii, F. taipaiensis, F. walujewii* |
|  |  | *rpl16-rps3* | 30 | *F. unibracteata* var*. wabuensis, F. unibracteata, F. hupehensis, F. pallidiflora, F. cirrhosa, F. taipaiensis, F. thunbergii* |
| 2 | Li et al., 2014.^[57]^ | *atpF* | 60 | *F. unibracteata* var*. wabuensis, F. thunbergii, F. walujewii, F. cirrhosa* |
|  |  | *petB* | 90 | *F. unibracteata* var*. wabuensis* |
|  |  | *psbZ* | 30 | *F. unibracteata* var*. wabuensis, F. unibracteata, F. hupehensis, F. cirrhosa, F. pallidiflora, F. thunbergii, F. walujewii* |
|  |  | *rpl16* | 60 | *F. unibracteata* var. *wabuensis, F. hupehensis, F. cirrhosa, F. thunbergii* |
|  |  | *rpoC2* | Because the length is too long (~ 4155 bp), give up evaluation of *rpoC2*. | |
|  |  | *rps16* | 70 | *F. unibracteata* var*. wabuensis, F. thunbergii, F. pallidiflora* |
|  |  | *rps19* | 40 | *F. unibracteata* var*. wabuensis, F. unibracteata, F. pallidiflora, F. walujewii, F. taipaiensis, F. walujewii* |
|  |  | *ycf3* | 70 | *F. unibracteata* var*. wabuensis, F. thunbergii, F. pallidiflora* |
| 3 | Li et al., 2016.^[58]^ | *ndhF-rpl32* | 40 | *F. unibracteata* var. *wabuensis, F. unibracteata, F. pallidiflora, F. walujewii, F. taipaiensis, F. walujewii* |
|  |  | *psaJ-rpl33* | 80 | *F. unibracteata* var. *wabuensis, F. unibracteata* |
|  |  | *rpoB-trnC* | 80 | *F. unibracteata* var*. wabuensis, F. unibracteata* |
|  |  | *trnD-trnY* | 60 | *F. unibracteata* var*. wabuensis, F. hupehensis, F. walujewii, F. thunbergii* |
|  |  | *trnE-trnT* | 70 | *F. unibracteata* var*. wabuensis, F. thunbergii, F. pallidiflora* |
|  |  | *trnP-psaJ* | 60 | *F. unibracteata* var. *wabuensis, F. unibracteata, F. hupehensis, F. pallidiflora* |
|  |  | *trnS-rps4* | 50 | *F. unibracteata* var. *wabuensis, F. unibracteata, F. walujewii, F. cirrhosa, F. thunbergii* |
| 4 | TÜRKTAŞ et al., 2012.^[59]^ | *trnL-trnF* | 40 | *F. unibracteata* var*. wabuensis, F. unibracteata, F. pallidiflora, F. walujewii, F. taipaiensis, F. walujewii* |
| 5 | Park et al., 2016.^[60]^ | *atpH-atpI* | 90 | *F. unibracteata* var*. wabuensis* |
|  |  | *atpI* | 30 | *F. unibracteata* var. *wabuensis, F. thunbergii, F. unibracteata, F. cirrhosa, F. taipaiensis, F. hupehensis, F. ussuriensis* |
|  |  | *ccsA* | 60 | *F. unibracteata* var*. wabuensis, F. unibracteata, F. hupehensis, F. pallidiflora* |
|  |  | *matK* | 60 | *F. unibracteata* var. *wabuensis, F. unibracteata, F. hupehensis, F. pallidiflora* |
|  |  | *ndhD* | 90 | *F. unibracteata* var. *wabuensis* |
|  |  | *ndhF* | 80 | *F. unibracteata* var. *wabuensis, F. unibracteata* |
|  |  | *psbK-psbI* | 30 | *F. unibracteata* var. *wabuensis, F. unibracteata, F. hupehensis, F. pallidiflora, F. cirrhosa, F. taipaiensis, F. thunbergii* |
|  |  | *psbM-trnD* | 70 | *F. unibracteata* var*. wabuensis, F. unibracteata, F. cirrhosa* |
|  |  | *rpoC1* | 70 | *F. unibracteata* var*. wabuensis, F. unibracteata, F. cirrhosa* |
|  |  | *rpoC2* | 60 | *F. unibracteata* var*. wabuensis, F. unibracteata, F. hupehensis, F. pallidiflora* |
|  |  | *rps16* | 70 | *F. unibracteata* var*. wabuensis, F. unibracteata, F. cirrhosa* |
|  |  | *rps16-trnQ* | 60 | *F. unibracteata* var*. wabuensis, F. unibracteata, F. hupehensis, F. pallidiflora* |
|  |  | *rps19* | 40 | *F. unibracteata* var. *wabuensis, F. unibracteata, F. hupehensis, F. pallidiflora, F. taipaiensis, F. thunbergii* |
|  |  | *trnE* | 20 | *F. unibracteata* var*. wabuensis, F. unibracteata, F. hupehensis, F. taipaiensis, F. pallidiflora, F. cirrhosa, F. ussuriensis, F. thunbergii* |
|  |  | *trnK* | 80 | *F. unibracteata* var*. wabuensis, F. unibracteata* |
|  |  | *trnK-rps16* | 80 | *F. unibracteata* var. *wabuensis, F. unibracteata* |
|  |  | *trnT* | 20 | *F. unibracteata* var*. wabuensis, F. unibracteata, F. hupehensis, F. taipaiensis, F. pallidiflora, F. cirrhosa, F. ussuriensis, F. thunbergii* |
|  |  | *trnY* | 20 | *F. unibracteata* var*. wabuensis, F. unibracteata, F. hupehensis, F. taipaiensis, F. pallidiflora, F. cirrhosa, F. ussuriensis, F. thunbergii* |
|  |  | *ycf1b* | 90 | *F. unibracteata* var*. wabuensis* |
|  |  | *ycf2* | 80 | *F. unibracteata* var. *wabuensis, F. unibracteata* |
|  |  | *ycf4-petD* | Because the length is too long (~ 15,650 bp), give up evaluation of *ycf4-petD*. | |
|  |  | *accD-psaI* | 60 | *F. unibracteata* var*. wabuensis, F. unibracteata, F. ussuriensis, F. thunbergii* |
| 6 | Bi et al., 2018.^[61]^ | *petA-psbJ* | 60 | *F. unibracteata* var. *wabuensis, F. unibracteata, F. thunbergii, F. taipaiensis* |
|  |  | *psbM-trnD* | 70 | *F. unibracteata* var. *wabuensis, F. unibracteata, F. thunbergii* |
|  |  | *rpl32-trnL* | 60 | *F. unibracteata* var. *wabuensis, F. unibracteata, F. thunbergii, F. taipaiensis* |
|  |  | *rpoB-petN* | 90 | *F. unibracteata* var*. wabuensis* |
|  |  | *rps11-rps8* | 40 | *F. unibracteata* var*. wabuensis, F. unibracteata, F. cirrhosa, F. hupehensis, F. taipaiensis, F. thunbergii* |
|  |  | *rps4-trnL* | 70 | *F. unibracteata* var*. wabuensis, F. unibracteata, F. thunbergii* |
|  |  | *trnK-rps16* | 80 | *F. unibracteata* var*. wabuensis, F. unibracteata* |
|  |  | *ycf1a* | 40 | *F. unibracteata, F. hupehensis, F. taipaiensis, F. cirrhosa, F. walujewii, F. thunbergii* |
|  |  | *ycf1b* | 90 | *F. unibracteata* var. *wabuensis* |
|  |  | *ycf4-cemA* | 80 | *F. unibracteata* var. *wabuensis, F. unibracteata* |
| 7 | Li et al., 2018.^[62]^ | *atpH-atpI* | 90 | *F. unibracteata* var*. wabuensis* |
|  |  | *matK-rps16* | 80 | *F. unibracteata* var*. wabuensis, F. unibracteata* |
|  |  | *petB* | 90 | *F. unibracteata* var. *wabuensis* |
|  |  | *petD-rpoA* | 20 | *F. unibracteata* var*. wabuensis, F. unibracteata, F. pallidiflora, F. walujewii, F. taipaiensis, F. hupehensis, F. thunbergii, F. cirrhosa* |
|  |  | *psbB-psbH* | 50 | *F. unibracteata* var*. wabuensis, F. unibracteata, F. pallidiflora, F. taipaiensis, F. thunbergii* |
|  |  | *rpl32-trnL* | 60 | *F. unibracteata* var*. wabuensis, F. unibracteata, F. cirrhosa, F. thunbergii* |
|  |  | *rps12-psbB* | 70 | *F. unibracteata* var*. wabuensis, F. pallidiflora, F. thunbergii* |
|  |  | *trnC-petN* | 60 | *F. unibracteata* var. *wabuensis, F. unibracteata, F. cirrhosa, F. thunbergii* |
|  |  | *trnE-psbT* | Because the length is too long (~ 42,209 bp), give up evaluation of *trnE-psbT*. | |
|  |  | *trnS-trnG* | 60 | *F. unibracteata* var*. wabuensis, F. unibracteata, F. hupehensis, F. thunbergii* |
|  |  | *trnT-trnF* | 70 | *F. unibracteata* var. *wabuensis, F. thunbergii, F. pallidiflora* |
|  |  | *ycf1b* | 90 | *F. unibracteata* var. *wabuensis* |
|  |  | *ycf2* | 90 | *F. unibracteata* var*. wabuensis* |
|  |  | *ycf4-cemA* | 80 | *F. unibracteata* var. *wabuensis, F. hupehensis* |
| 8 | Moon et al., 2018.^[63]^ | *matK* | 60 | *F. unibracteata* var*. wabuensis, F. unibracteata, F. hupehensis, F. thunbergii* |
|  |  | *rps16* | 70 | *F. unibracteata* var. *wabuensis, F. thunbergii, F. pallidiflora* |
| 9 | Lu et al., 2021.^[64]^ | *ndhE-ndhG* | 50 | *F. unibracteata* var*. wabuensis, F. unibracteata, F. hupehensis, F. pallidiflora, F. thunbergii* |
|  |  | *ndhG-ndhI* | 60 | *F. unibracteata* var*. wabuensis, F. unibracteata, F. hupehensis, F. pallidiflora* |
|  |  | *petN-psbM* | 30 | *F. unibracteata* var*. wabuensis, F. unibracteata, F. hupehensis, F. pallidiflora, F. cirrhosa, F. taipaiensis, F. thunbergii* |
|  |  | *rbcL* | 70 | *F. unibracteata* var*. wabuensis, F. unibracteata, F. cirrhosa* |
|  |  | *rpl16-rps3* | 30 | *F. unibracteata* var. *wabuensis, F. unibracteata, F. hupehensis, F. pallidiflora, F. cirrhosa, F. taipaiensis, F. thunbergii* |
|  |  | *rpl32-trnL* | 60 | *F. unibracteata* var*. wabuensis, F. unibracteata, F. cirrhosa, F. thunbergii* |
|  |  | *rpoB-trnC* | 80 | *F. unibracteata* var*. wabuensis, F. unibracteata* |
|  |  | *rps2-rpoC2* | 60 | *F. unibracteata* var. *wabuensis, F. unibracteata, F. hupehensis, F. pallidiflora* |
|  |  | *trnK-rps16* | 80 | *F. unibracteata* var. *wabuensis, F. unibracteata* |
|  |  | *trnS-trnG* | 60 | *F. unibracteata* var. *wabuensis, F. unibracteata, F. hupehensis, F. pallidiflora* |
|  |  | *trnT-trnL* | 80 | *F. unibracteata* var*. wabuensis, F. unibracteata* |
| 10 | Chen et al., 2019.^[65]^ | *atpH-atpI* | 90 | *F. thunbergii* |
|  |  | *petN-psbM* | 30 | *F. ussuriensis, F. unibracteata, F. pallidiflora, F. taipaiensis, F. hupehensis, F. thunbergii, F. cirrhosa* |
|  |  | *psbE-petL* | 80 | *F. unibracteata* var*. wabuensis, F. unibracteata* |
|  |  | *psbM-trnD* | 70 | *F. unibracteata* var. *wabuensis, F. thunbergii, F. pallidiflora* |
|  |  | *rpoB-trnC* | 80 | *F. unibracteata* var. *wabuensis, F. unibracteata* |
|  |  | *trnS-rps4* | 50 | *F. unibracteata* var. *wabuensis, F. unibracteata, F. walujewii, F. cirrhosa, F. thunbergii* |
|  |  | *trnT-psbD* | 80 | *F. unibracteata* var*. wabuensis, F. unibracteata* |
|  |  | *trnT-trnL* | 80 | *F. unibracteata* var*. wabuensis, F. unibracteata* |
|  |  | *ycf1b* | 90 | *F. unibracteata* var. *wabuensis* |
|  |  | *ycf4-cemA* | 80 | *F. unibracteata* var*. wabuensis, F. thunbergii* |
|  |  | *matK* | 60 | *F. unibracteata* var*. wabuensis, F. unibracteata, F. hupehensis, F. pallidiflora* |

*The species resolutions were calculated using tree-based method.
