# Supplementary material for: Plant super-barcode: a case study on genome-based identification for closely related species of Fritillaria
Source: Chin Med. 2021 Jul 5;16:52. doi: 10.1186/s13020-021-00460-z (PMC8256587; doi:10.1186/s13020-021-00460-z)
Supplement: Supplementary file 5 — Additional file 5: Figure S2. Phylogenetic relationships among the ten Fritillaria species based on complete cp genome sequences by the maximum likelihood (ML) method. Lilium brownie and Cardiocrinum giganteum were set as the compound outgroups. [file 13020_2021_460_MOESM5_ESM.docx]

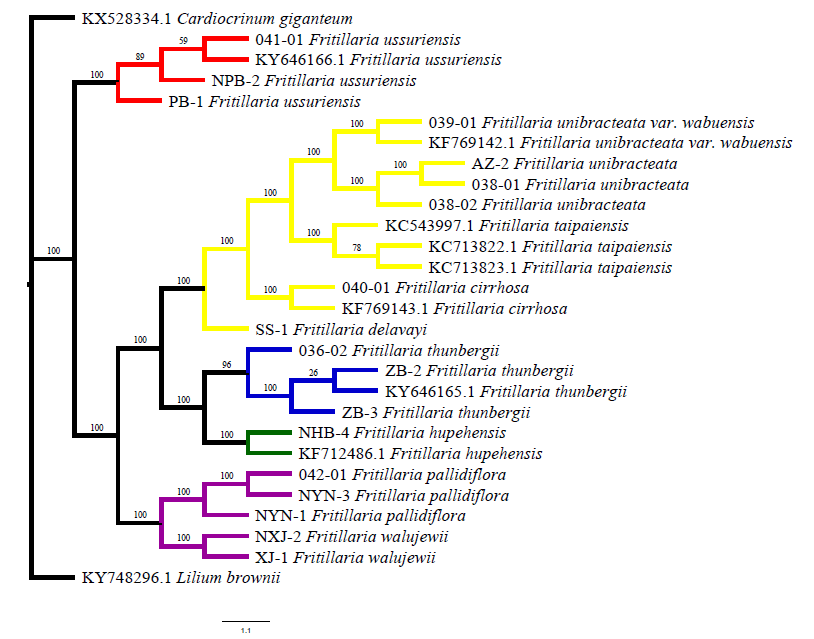
**Additional file 5: Figure S2.** Phylogenetic relationships among the ten *Fritillaria* species based on complete cp genome sequences by the maximum likelihood (ML) method. *Lilium brownie* and *Cardiocrinum giganteum* were set as the compound outgroups.
