# Supplementary figures and images for: Plant super-barcode: a case study on genome-based identification for closely related species of Fritillaria
Source: Chin Med. 2021 Jul 5;16:52. doi: 10.1186/s13020-021-00460-z (PMC8256587; doi:10.1186/s13020-021-00460-z)

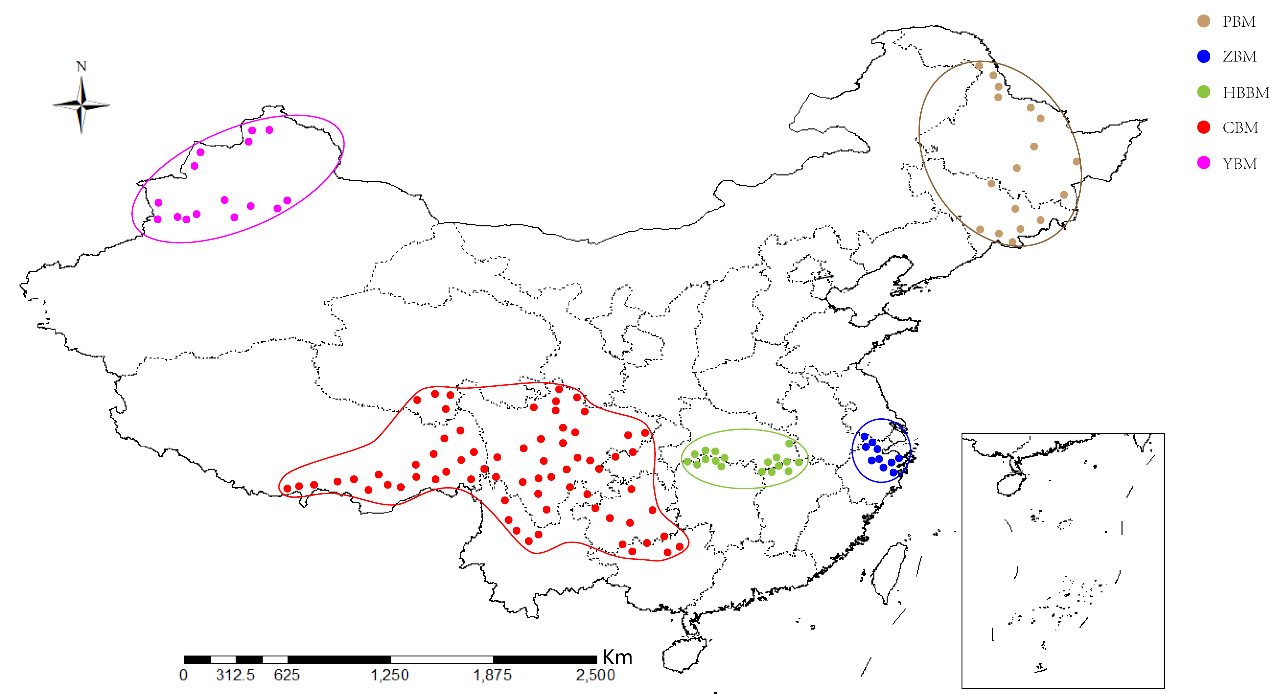


**Additional file 6: Figure S3.** Ecological and geographical regions of five BM material medica.

Supplement: Supplementary file 6 — Additional file 6: Figure S3. Ecological and geographical regions of five BM material medica. [file 13020_2021_460_MOESM6_ESM.docx]
