# Supplementary material for: Plant super-barcode: a case study on genome-based identification for closely related species of Fritillaria
Source: Chin Med. 2021 Jul 5;16:52. doi: 10.1186/s13020-021-00460-z (PMC8256587; doi:10.1186/s13020-021-00460-z)
Supplement: Supplementary file 7 — Additional file 7: Verification of discrimination ability of 57 highly variable loci selected by published works. [file 13020_2021_460_MOESM7_ESM.docx]

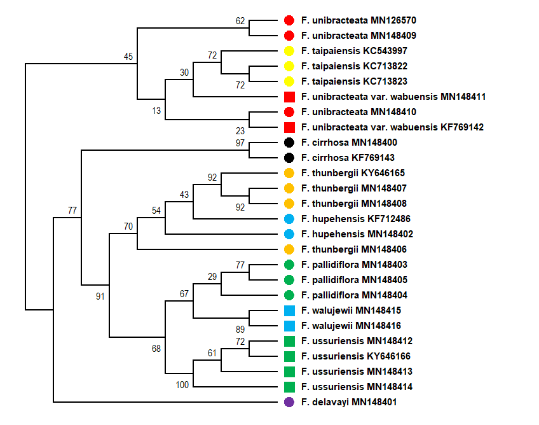


**1**. NJ tree of accD-psal regions from 26 individuals from ten *Fritillaria* species.


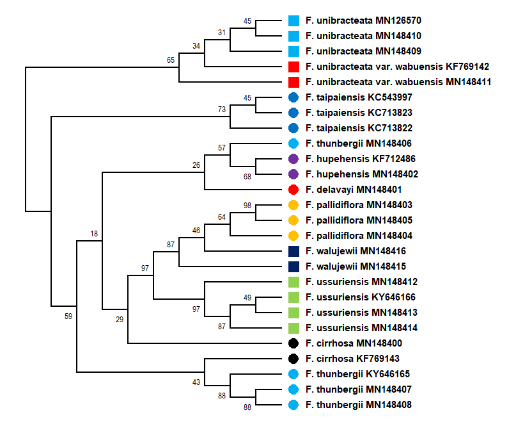


**2**. NJ tree of atpF regions from 26 individuals from ten *Fritillaria* species.


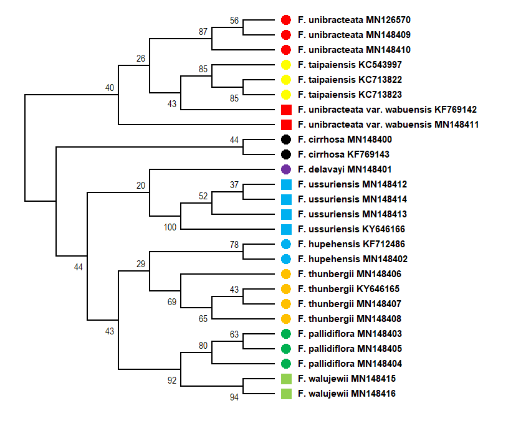


**3**. NJ tree of atpH-atpl regions from 26 individuals from ten *Fritillaria* species.


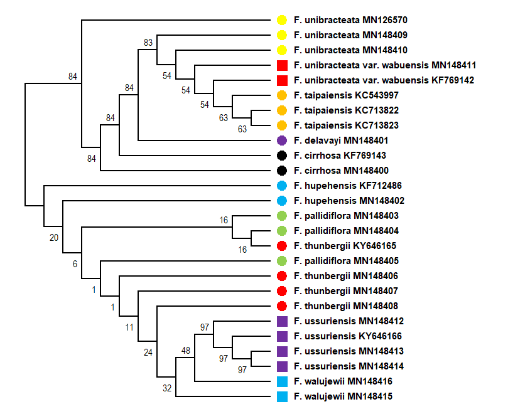


**4**. NJ tree of atpl regions from 26 individuals from ten *Fritillaria* species.


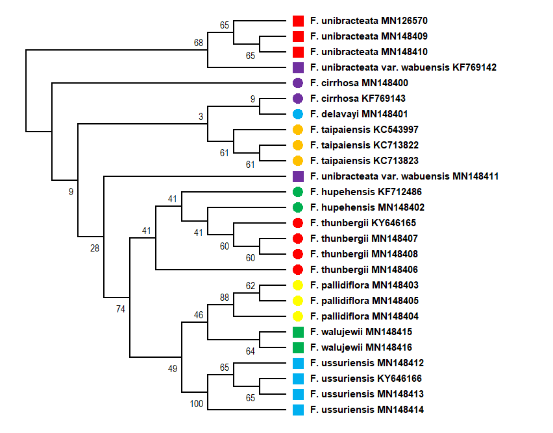
**5**. NJ tree of ccsA regions from 26 individuals from ten *Fritillaria* species.


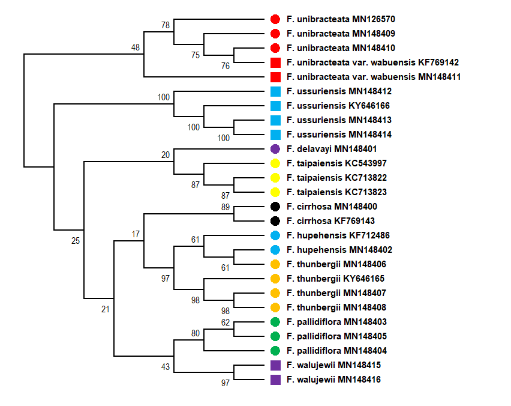


**6**. NJ tree of matK regions from 26 individuals from ten *Fritillaria* species.


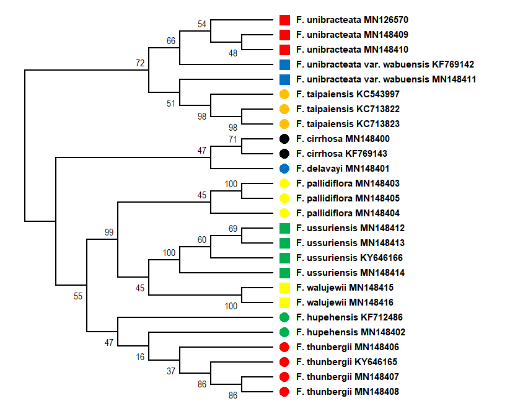


**7**. NJ tree of matK-rps16 regions from 26 individuals from ten *Fritillaria* species.


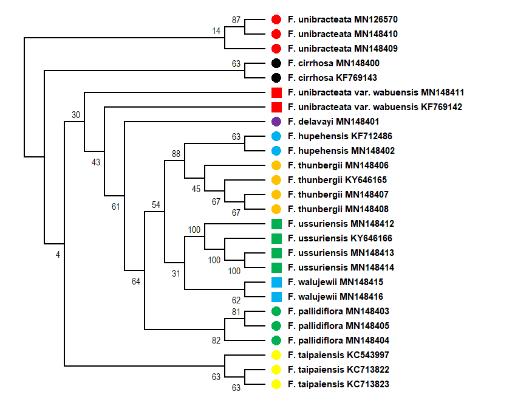


**8**. NJ tree of ndhD regions from 26 individuals from ten *Fritillaria* species.


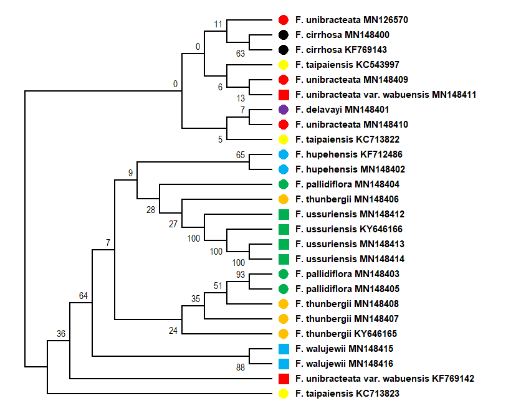


**9**. NJ tree of ndhE-ndhG regions from 26 individuals from ten *Fritillaria* species.


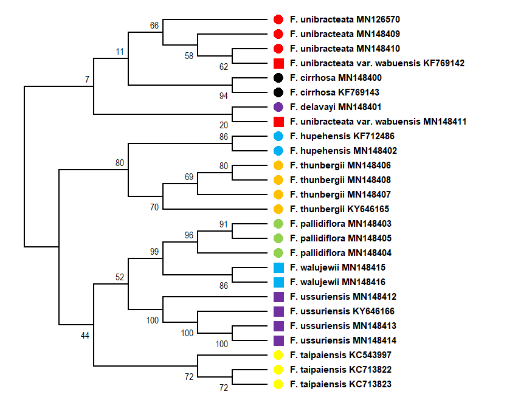


**10**. NJ tree of ndhF regions from 26 individuals from ten *Fritillaria* species.


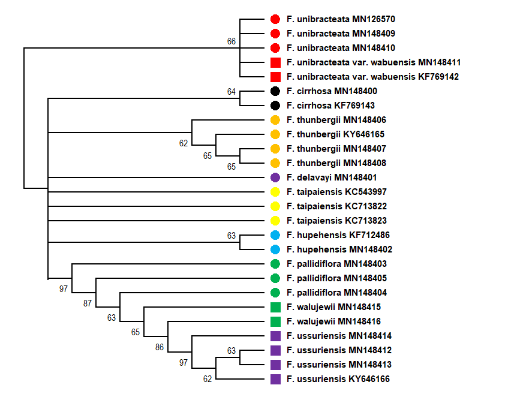


**11**. NJ tree of ndhF-rpl32 regions from 26 individuals from ten *Fritillaria* species.


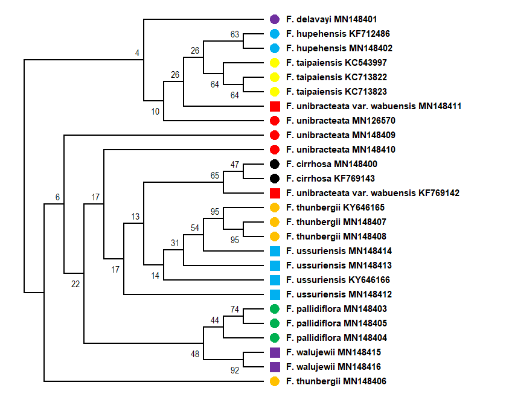


**12**. NJ tree of ndhG-ndhl regions from 26 individuals from ten *Fritillaria* species.


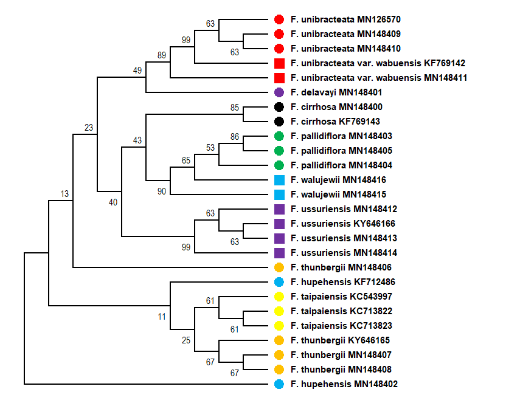


**13**. NJ tree of petA-psbL regions from 26 individuals from ten *Fritillaria* species.
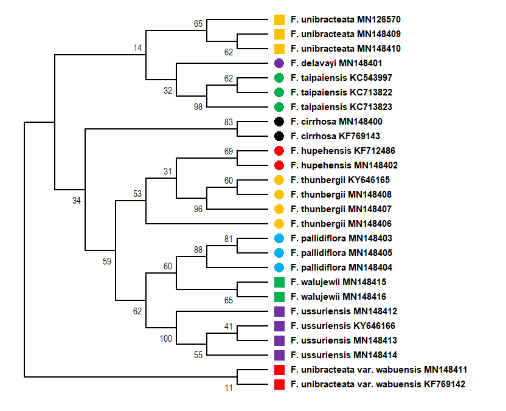
**14**. NJ tree of petB regions from 26 individuals from ten *Fritillaria* species.
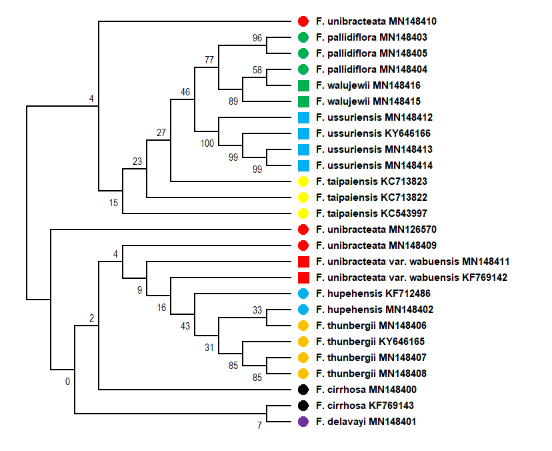
**15**. NJ tree of petD-rpoA regions from 26 individuals from ten *Fritillaria* species.
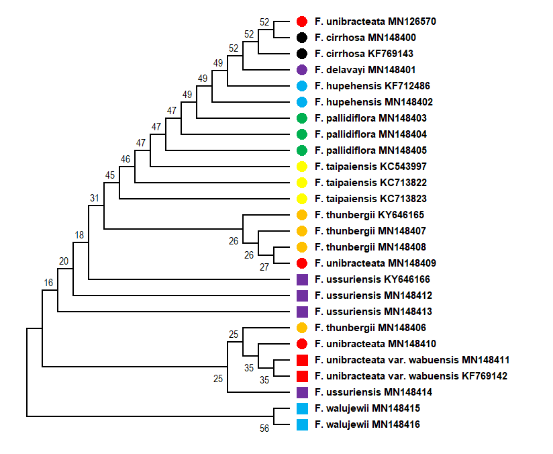
**16**. NJ tree of petN-psbM regions from 26 individuals from ten *Fritillaria* species.


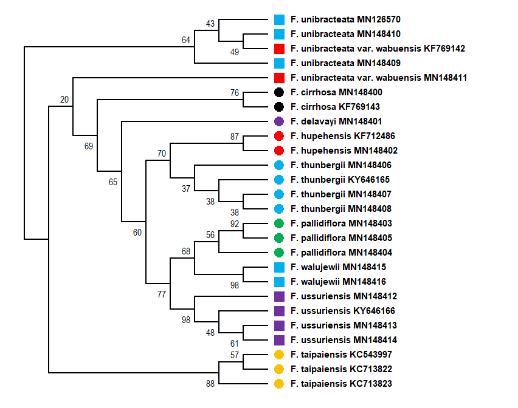


**17**. NJ tree of psaJ-rpl33 regions from 26 individuals from ten *Fritillaria* species.
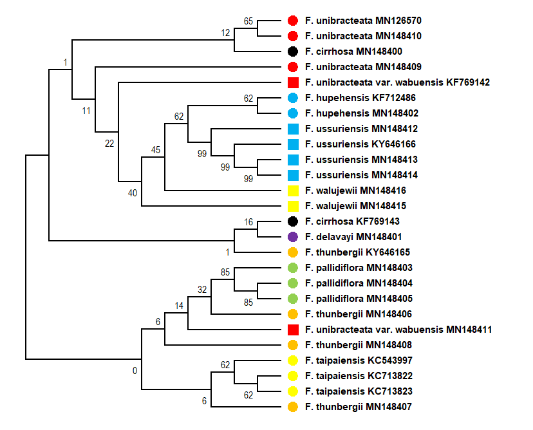
**18**. NJ tree of psbB-psbH regions from 26 individuals from ten *Fritillaria* species.
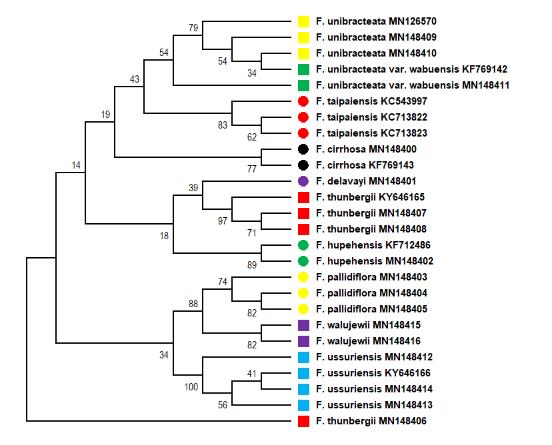
**19**. NJ tree of psbE-petL regions from 26 individuals from ten *Fritillaria* species.
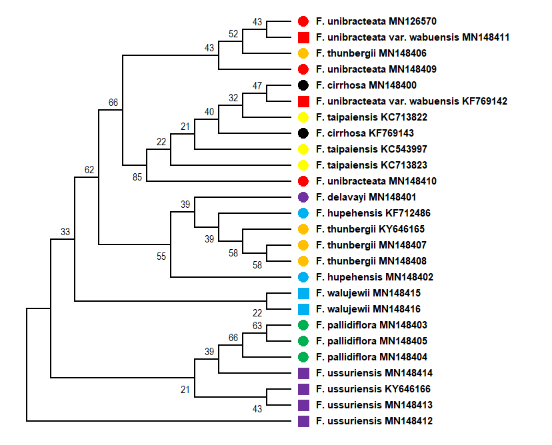
**20**. NJ tree of psbK-psbl regions from 26 individuals from ten *Fritillaria* species.


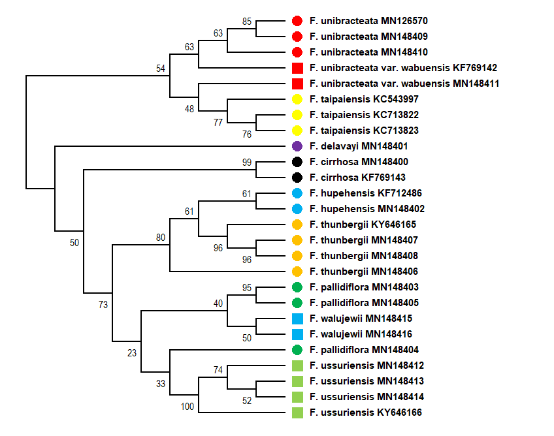
**21**. NJ tree of psbM-trnD regions from 26 individuals from ten *Fritillaria* species.


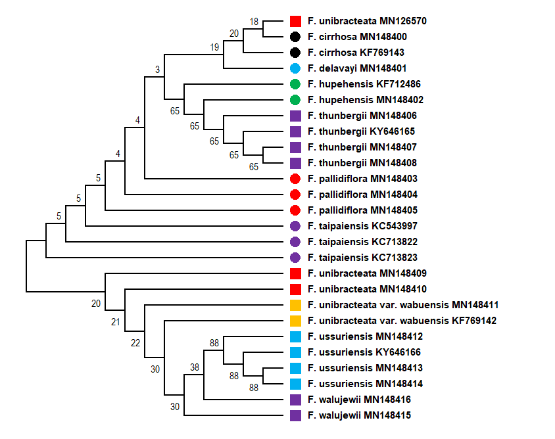
**22**. NJ tree of psbZ regions from 26 individuals from ten *Fritillaria* species.


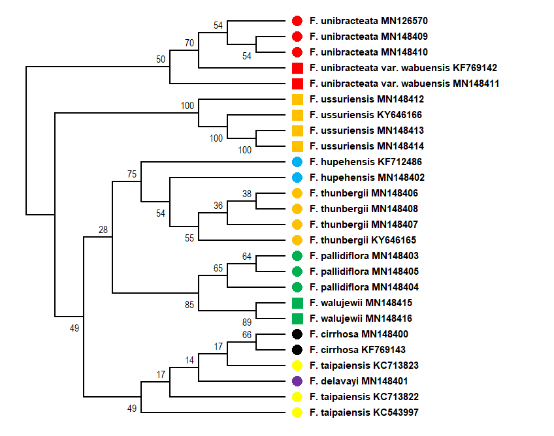
**23**. NJ tree of rbcL regions from 26 individuals from ten *Fritillaria* species.


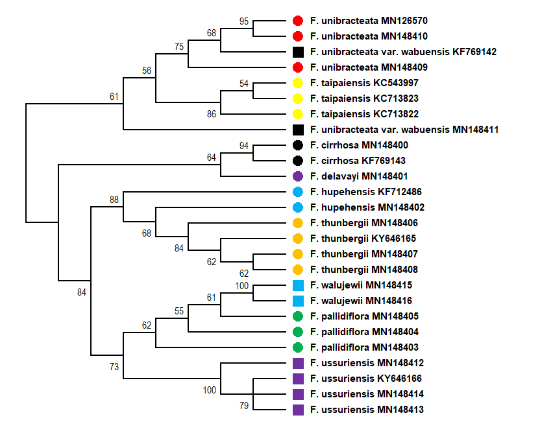
**24**. NJ tree of rpl16 regions from 26 individuals from ten *Fritillaria* species.
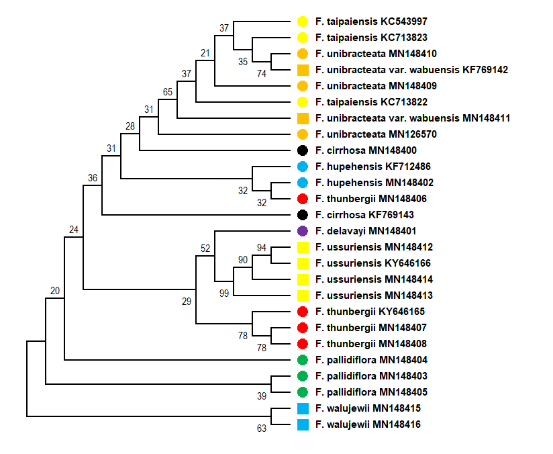


**25**. NJ tree of rpl16-rps3 regions from 26 individuals from ten *Fritillaria* species.


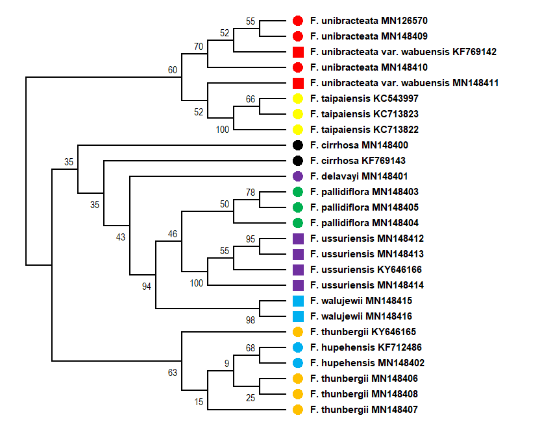


**26**. NJ tree of rpl32-trnL regions from 26 individuals from ten *Fritillaria* species.


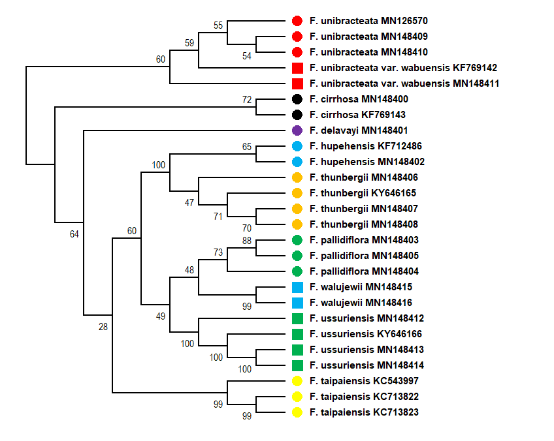


**27**. NJ tree of rpoB-petN regions from 26 individuals from ten *Fritillaria* species.


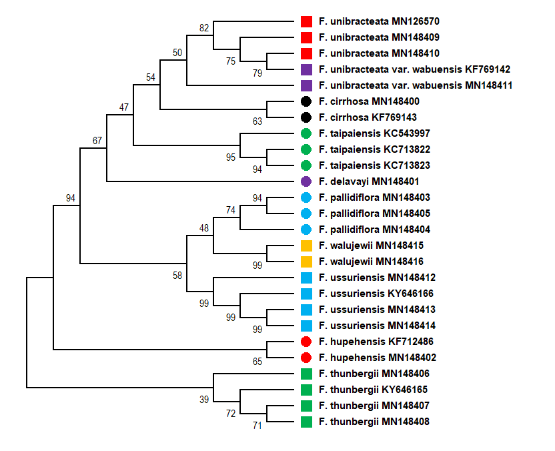
**28**. NJ tree of rpoB-trnC regions from 26 individuals from ten *Fritillaria* species.


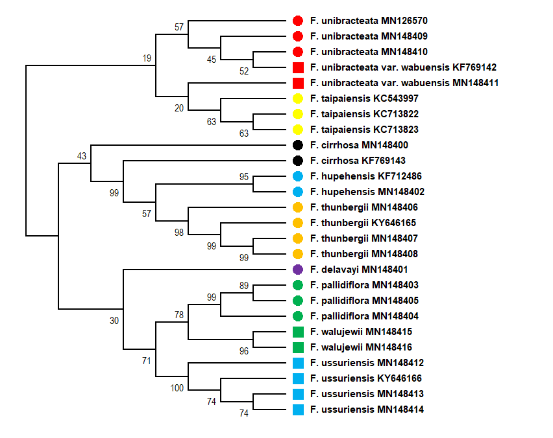


**29**. NJ tree of rpoC1 regions from 26 individuals from ten *Fritillaria* species.


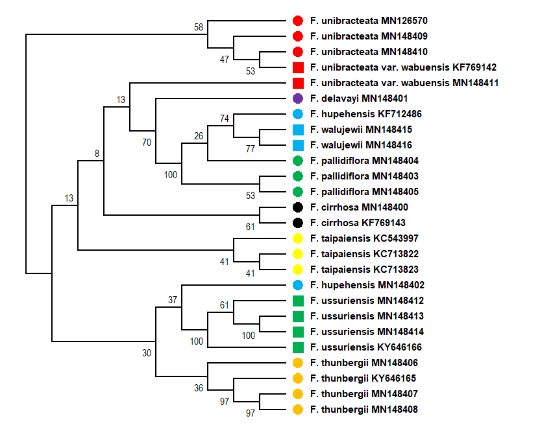


**30**. NJ tree of rpoC2 regions from 26 individuals from ten *Fritillaria* species.


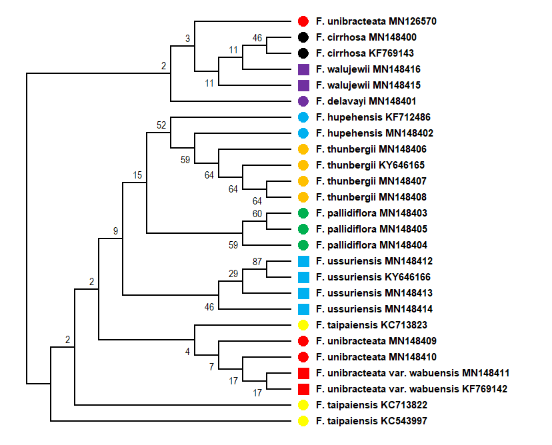
**31**. NJ tree of rps2-rpoC2 regions from 26 individuals from ten *Fritillaria* species.


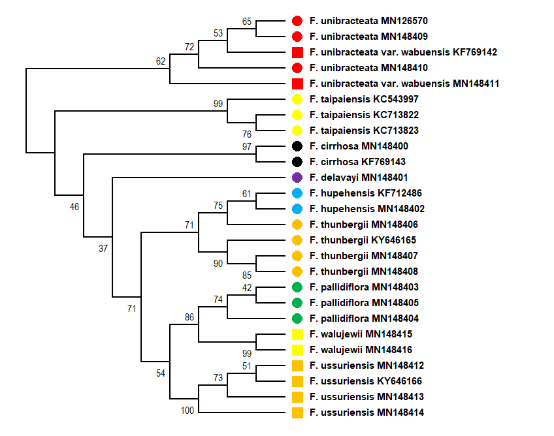


**32**. NJ tree of rps4-trnL regions from 26 individuals from ten *Fritillaria* species.


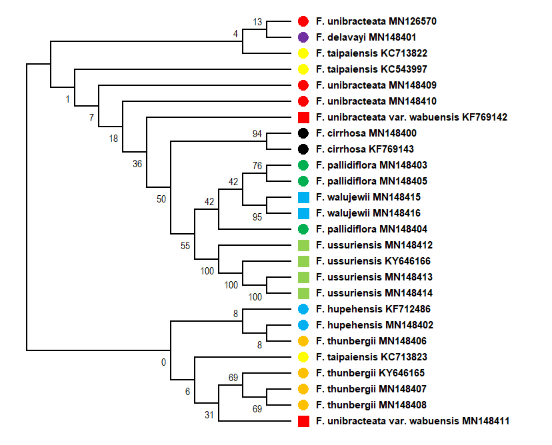


**33**. NJ tree of rps11-rps8 regions from 26 individuals from ten *Fritillaria* species.
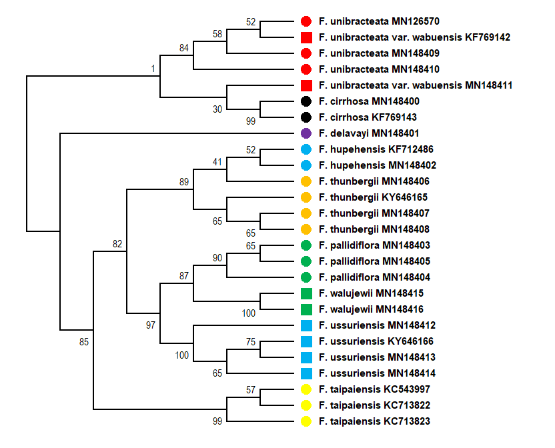


**34**. NJ tree of rps12-psbB regions from 26 individuals from ten *Fritillaria* species.


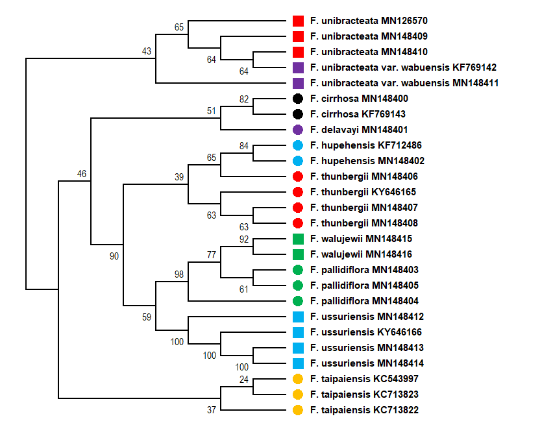
**35**. NJ tree of rps16 regions from 26 individuals from ten *Fritillaria* species.


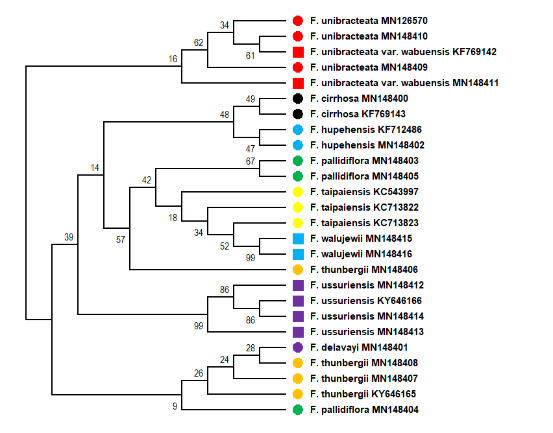
**36**. NJ tree of rps16-trnQ regions from 26 individuals from ten *Fritillaria* species.


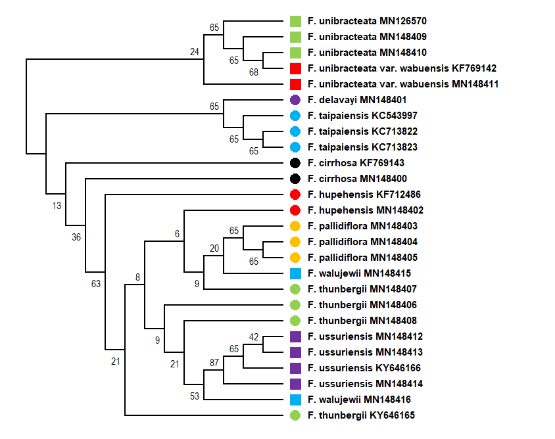


**37**. NJ tree of rps19 regions from 26 individuals from ten *Fritillaria* species.
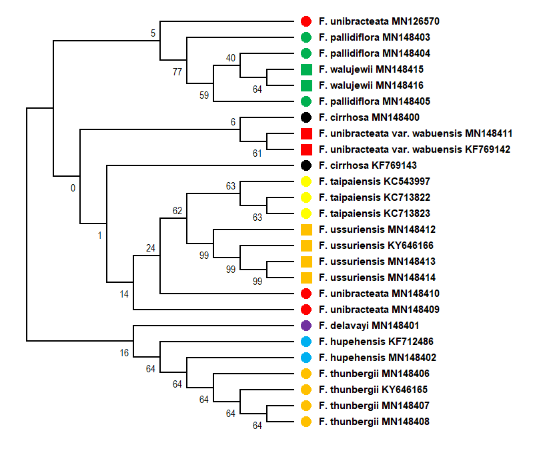
**38**. NJ tree of trnC-petN regions from 26 individuals from ten *Fritillaria* species.


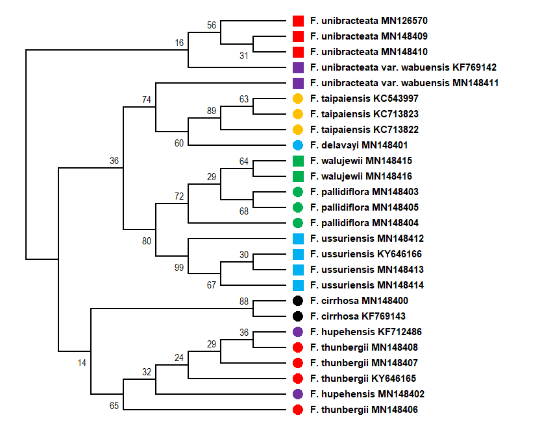


**39**. NJ tree of trnD-trnY regions from 26 individuals from ten *Fritillaria* species.


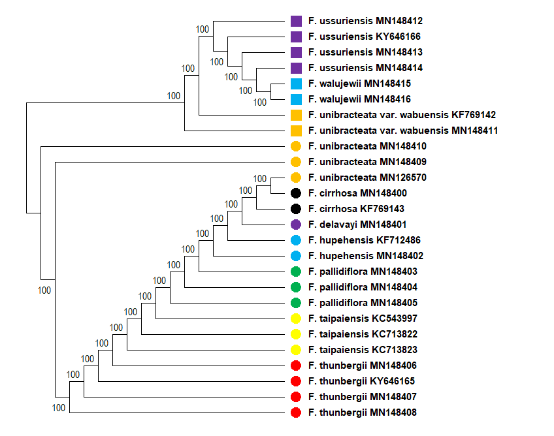
**40**. NJ tree of trnE regions from 26 individuals from ten *Fritillaria* species.
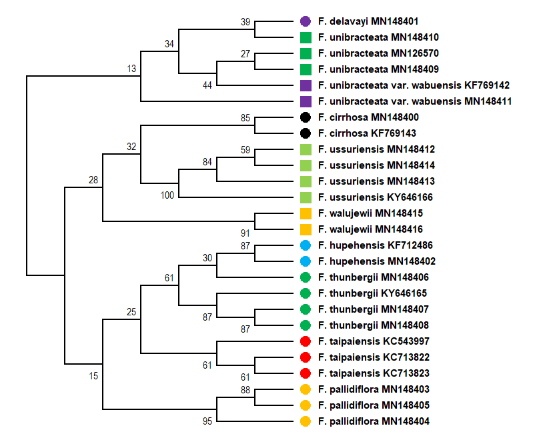


**41**. NJ tree of trnE-trnT regions from 26 individuals from ten *Fritillaria* species.


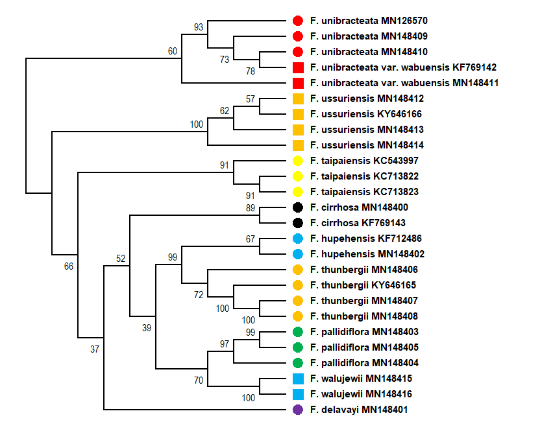
**42**. NJ tree of trnK regions from 26 individuals from ten *Fritillaria* species.


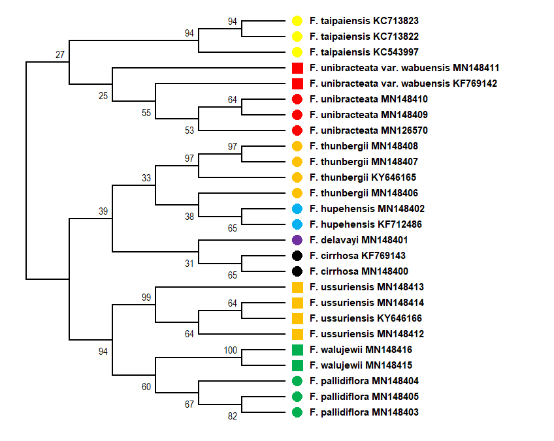


**43**. NJ tree of trnK-rps16 regions from 26 individuals from ten *Fritillaria* species.
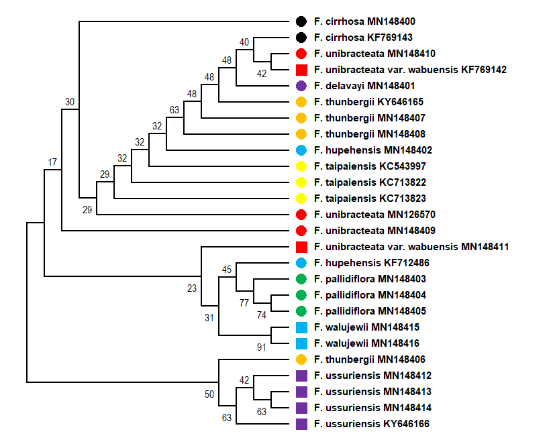
 **44**. NJ tree of trnL-trnF regions from 26 individuals from ten *Fritillaria* species.


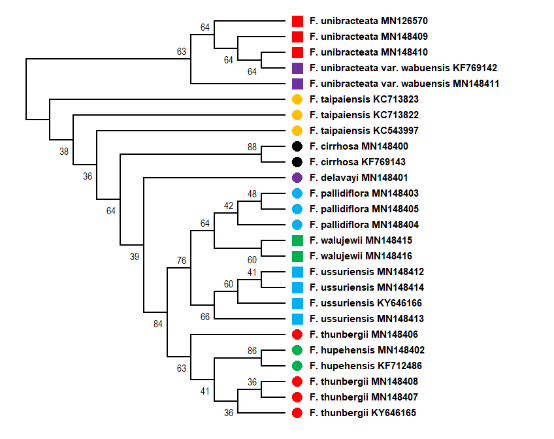


**45**. NJ tree of trnP-psaJ regions from 26 individuals from ten *Fritillaria* species.


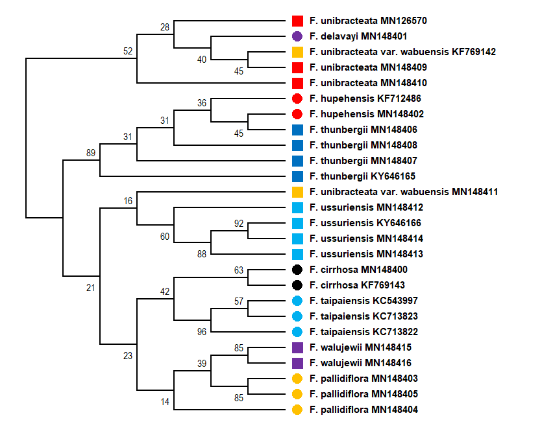
**46**. NJ tree of trnS-rps4 regions from 26 individuals from ten *Fritillaria* species.


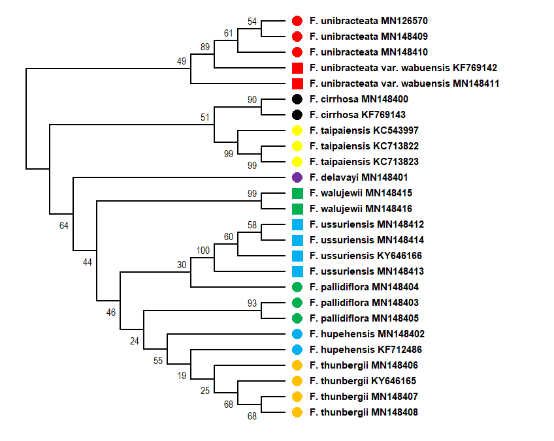
**47**. NJ tree of trnS-trnG regions from 26 individuals from ten *Fritillaria* species.
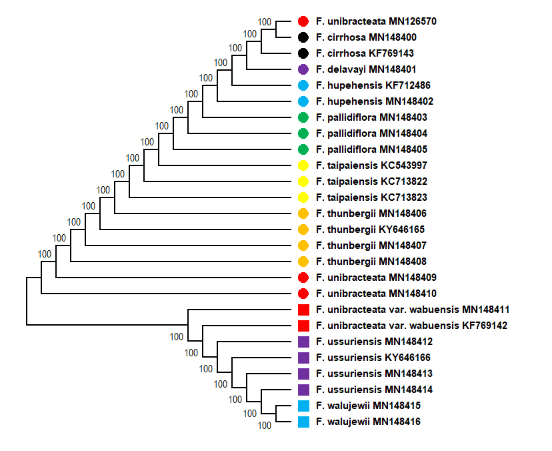


**48**. NJ tree of trnT regions from 26 individuals from ten *Fritillaria* species.


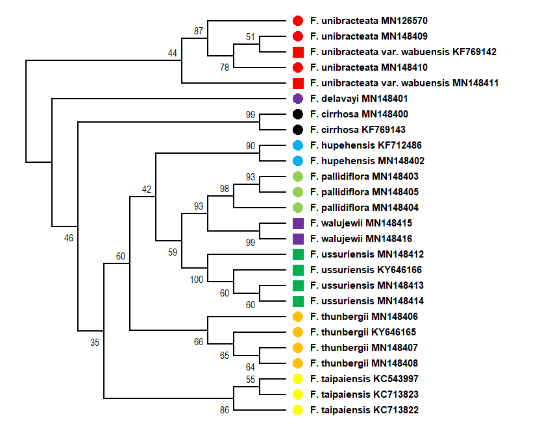
**49**. NJ tree of trnT-psbD regions from 26 individuals from ten *Fritillaria* species.
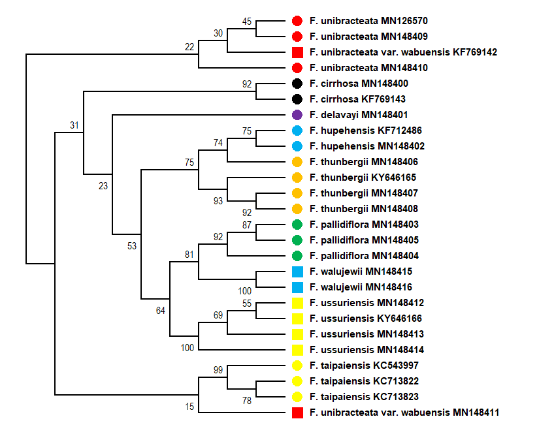
**50**. NJ tree of trnT-trnF regions from 26 individuals from ten *Fritillaria* species.
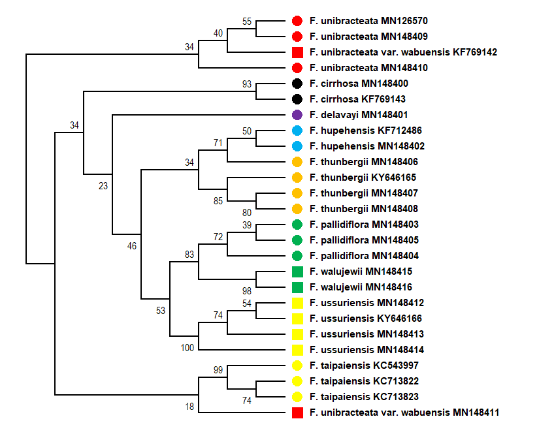
**51**. NJ tree of trnT-trnL regions from 26 individuals from ten *Fritillaria* species.
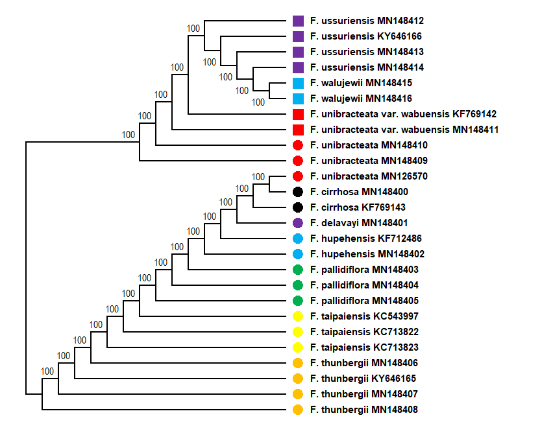
**52**. NJ tree of trnY regions from 26 individuals from ten *Fritillaria* species.
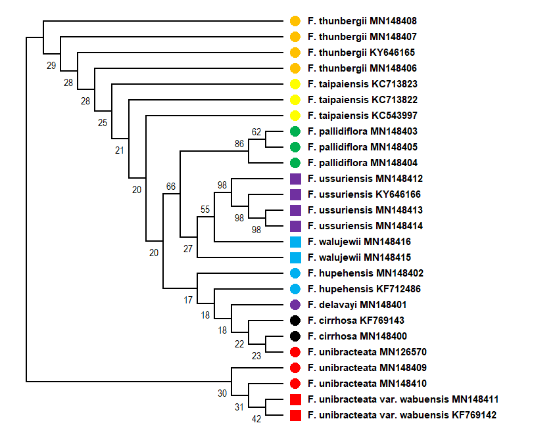
**53**. NJ tree of ycf1a regions from 26 individuals from ten *Fritillaria* species.


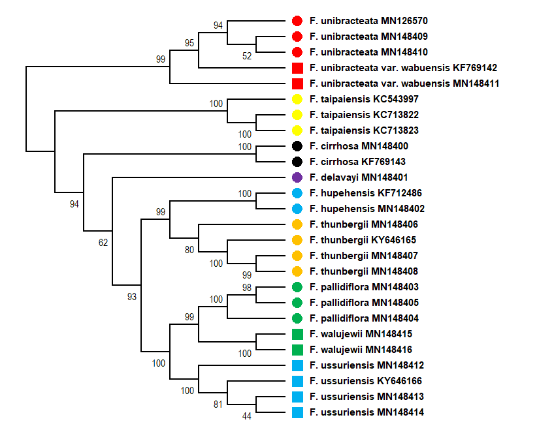
**54**. NJ tree of ycf1b regions from 26 individuals from ten *Fritillaria* species.
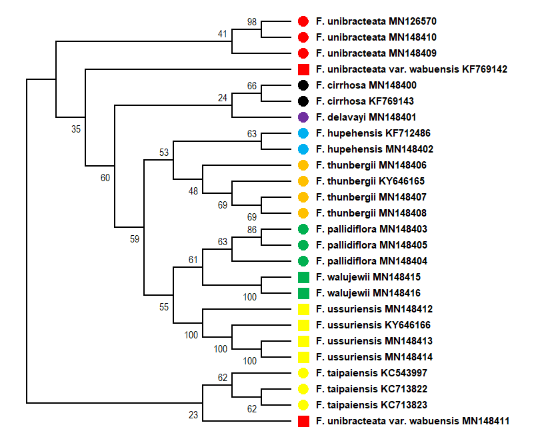
**55**. NJ tree of ycf2 regions from 26 individuals from ten *Fritillaria* species.


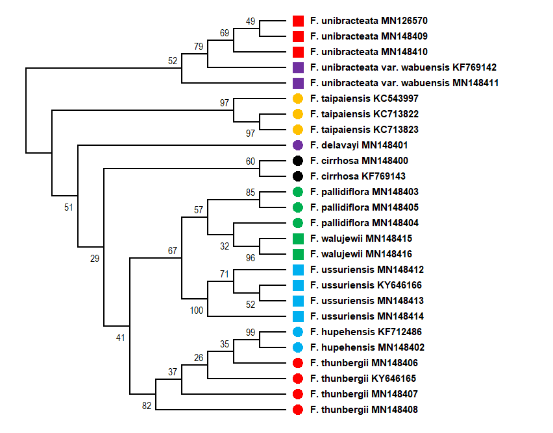
**56**. NJ tree of ycf3 regions from 26 individuals from ten *Fritillaria* species.


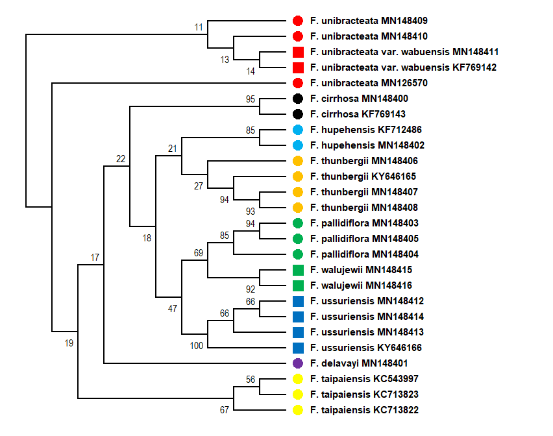
**57**. NJ tree of ycf4-cemA regions from 26 individuals from ten *Fritillaria* species.

**Additional file 7：**Verification of discrimination ability of 57 highly variable loci selected by published works.
